# A clinical experience-based Chinese herbal formula improves ethanol-induced drunken behavior and hepatic steatohepatitis in mice models

**DOI:** 10.1186/s13020-023-00753-5

**Published:** 2023-05-01

**Authors:** Han Gao, Zhen Li, Yao Liu, Yong-kang Zhao, Cheng Cheng, Feng Qiu, Yuan Gao, Ya-wen Lu, Xin-hua Song, Jia-bo Wang, Zhi-tao Ma

**Affiliations:** 1 College of Pharmacy, Fujian University of Traditional Chinese Medicine, Fuzhou, 350122 Fujian China; 2 School of Traditional Chinese Medicine, Capital Medical University, Beijing, 100069 China; 3 Department of Hepatology, Fifth Medical Center of Chinese, PLA General Hospital, Beijing, 100039 China; 4 College of Pharmacy, Henan University of Traditional Chinese Medicine, Henan, 450046 Zhengzhou China; 5 Department of Infectious Disease, Beijing Hospital of Traditional Chinese Medicine, Capital Medical University, Beijing, 100010 China; 6 College of Pharmacy, Chengdu University of Traditional Chinese Medicine, Chengdu, 611137 China; 7 Department of Pharmacy, Jincheng General Hospital, Jincheng, 048006 Shanxi China

**Keywords:** Traditional Chinese medicine, Alcoholic liver disease, Steatosis, Inflammation, Ethanol metabolism, Drunken behavior

## Abstract

**Background:**

Bao-Gan-Xing-Jiu-Wan (BGXJW) is a clinical experience-based Chinese herbal formula. Its efficacy, pharmacological safety, targeted function, process quality, and other aspects have met the evaluation standards and the latest requirements of preparations. It could prevent and alleviate the symptoms of drunkenness and alcoholic liver injury clinically. The present work aims to elucidate whether BGXJW could protect against drunkenness and alcoholic liver disease in mice and explore the associated mechanism.

**Material and methods:**

We used acute-on-chronic (NIAAA) mice model to induce alcoholic steatosis, and alcohol binge-drinking model to reappear the drunk condition. BGXJW at indicated doses were administered by oral gavage respectively to analyze its effects on alcoholic liver injury and the associated molecular mechanisms.

**Results:**

BGXJW had no cardiac, hepatic, renal, or intestinal toxicity in mice. Alcoholic liver injury and steatosis in the NIAAA mode were effectively prevented by BGXJW treatment. BGXJW increased the expression of alcohol metabolizing enzymes ADH, CYP2E1, and ALDH2 to enhance alcohol metabolism, inhibited steatosis through regulating lipid metabolism, counteracted alcohol-induced upregulation of lipid synthesis related proteins SREBP1, FASN, and SCD1, meanwhile it enhanced fatty acids β-oxidation related proteins PPAR-α and CPT1A. Alcohol taken enhanced pro-inflammatory TNF-α, IL-6 and down-regulated the anti-inflammatory IL-10 expression in the liver, which were also reversed by BGXJW administration. Moreover, BGXJW significantly decreased the blood ethanol concentration and alleviated drunkenness in the alcohol binge-drinking mice model.

**Conclusions:**

BGXJW could effectively relieve drunkenness and prevent alcoholic liver disease by regulating lipid metabolism, inflammatory response, and alcohol metabolism.

**Graphical Abstract:**

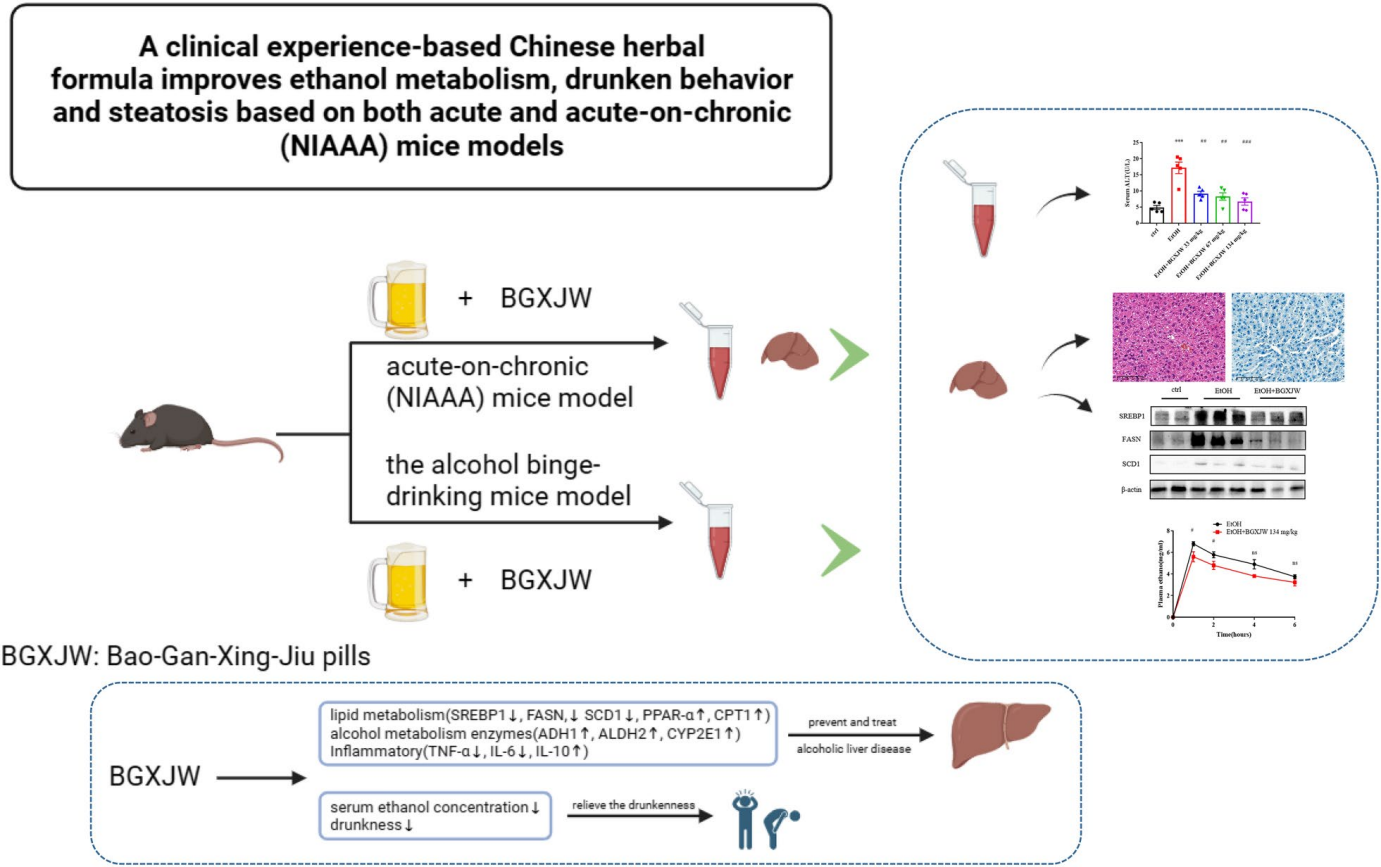

**Supplementary Information:**

The online version contains supplementary material available at 10.1186/s13020-023-00753-5.

## Introduction

With the development of the economy and society, the consumption of alcohol keeps increasing, and the incidence of alcoholic liver disease (ALD), one of the most common preventable liver diseases induced by long time or heavy alcohol intake, is on the rise and brings a huge disease burden to the world economy [38]. In China, there has been a consistent net increase in alcohol consumption since 1990, i.e., the number of heavy drinkers increased from 0.21% in 1982 to 14.8% in 2000, and the ALD prevalence increased from 2.27% in 2000 to 8.74% in 2015 [38, 54, 55]. The ALD patients in China, estimated to be at least 62 million people, account for 4.5% of the world's total in 2017 [56]. The associated pathological features are from early steatosis to alcoholic fatty liver (AFLD), hepatitis, liver fibrosis, cirrhosis, and eventually hepatocellular carcinoma [30, 37, 44]. Nevertheless, even before pathological alteration occurs, both acute and chronic alcohol consumption highly affects daily activities. Detailly, drunkenness induced by alcohol binge drinking shows uncoordinated limb movement, impaired thinking, and decision-making capability, and is often accompanied by symptoms such as headache, thirst, and unconsciousness even when sober from a hangover[6, 17, 22, 40, 49, 53]. It is well recognized that alcohol intake, either acute or chronic, both affects life quality and human health as it could lead to ALD. However, up till the present moment, there is no consensus anti-alcoholic drug, and the most effective treatment for ALD remains abstinence, other treatments such as nutritional treatment, drug treatment, psychotherapy, and liver transplantation are also applied clinically based on the stage of ALD [19, 24, 48], while these may not always be practical or sufficient [57]. Given the situation, it is very crucial to explore a novel therapy for the prevention or treatment of ALD.

Traditional Chinese medicine (TCM) plays an important role in the prevention and treatment of ALD through the synergistic effects of its various components as many TCM formulas had been reported to be effective in protecting against both various discomfort feelings by alcohol binge drinking and alcohol-induced liver injury by chronic alcohol taken [13, 16, 28]. Among them, Bao-Gan-Xing-Jiu-Wan (BGXJW), a clinical experience-based Chinese herbal preparation (pills), exhibit excellent clinical outcomes as they can effectively prevent ALD and relieve drunkenness. However, its scientific mechanism is still unclear, which limits the application of BGXJW on a larger scale.

Here we studied the pharmacological phenotypes of BGXJW in the alcohol binge-drinking mice model and acute-on-chronic (NIAAA) model [2] along with the associated mechanisms. The results showed that BGXJW can effectively treat ALD by regulating lipid synthesis, fatty acid β-oxidation, inflammation, and alcohol metabolism. In addition, BGXJW can also effectively alleviate drunkenness, which may have an important relationship with its role in reducing blood ethanol concentration.

## Materials and methods

### BGXJW preparation

BGXJW powder was provided by Hebei Traditional Chinese medicine liver disease hospital. It is the extracts from the roots of *Pueraria lobata* (Willd.) Ohwi (Gegen), flos of *Pueraria lobata* (Willd.) Ohwi (Gehua), seeds of *Hovenia Dulcis* Thunb. (Zhijuzi), seeds of *Cassia abbreviata* Oliv. (Juemingzi), flos of *Chrysanthemum abolinii* (Kovalevsk.) H.Ohashi and Yonek. (Juhua), rhizomes of *Alpinia officinarum* Hance (Gaoliangjiang), roots of *Salvia miltiorrhiza* Bunge (Danshen), fruits of *Morus alba* L. (Sangshen), fruits of *Amomum tsao-ko* Crevost *et* Lemarie (Caoguo). The doses of these herbs are in fixed proportions.

To investigate BGXJW’s effects and the associated mechanisms, the BGXJW powder was added into 0.9% saline and ultrasounded to promote dissolution.

### Reagents and instruments

#### Reagents

Buspirone hydrochloride (purity 98%, TRC, Toronto, Canada); phenytoin sodium(purity 99.97%, Bidepharm, Shanghai, China); Rodent Liquid Diet Lieber-DeCarli ’82, Ethanol Shake and Pour 4 Liters/Bag(Bio-serv, New Jersey, USA); Rodent Liquid Diet Lieber-DeCarli ’82, Control Shake and Pour 4 Liters/Bag( Bio-serv, New Jersey, USA); The ALT, AST, TG, T-CHO, CK-MB and CK-MM kits (96 T, Nanjing Jiancheng Bioengineering Institute, NanJing, China. TNF-α, IL-6, and IL-10 ELISA kits(96 T, CLOUD-CLONE CORP. Wuhan, China);liver tissues was isolated with FastPure Cell/Tissue Total RNA Isolation Kit (50 T, Vazyme, Nanjing, China) RIPA Lysis Buffer were purchased from Biorigin, Beijing, China; polyvinylidene fluoride membrane were purchased from MILLIPORE, Massachusetts, USA; The antibodies used against CYP2E1, SCD1, FASN, and PPAR-α were purchased from Abcam, Massachusetts, USA; antibodies against ADH1 and ALDH2 were purchased from Boster, California, USA and Cell Signaling Technology, Boston, USA separately; Antibodies against SREBP-1 (A-4) were purchased from Santa Cruz Biotechnology,Santa Cruz, USA; Antibodies against β-Actin were purchased from Bioworld, Minnesota, USA; HRP-Goat Anti-Rabbit IgG(H + L) was purchased from Biorigin, Beijing, China; Goat Anti-Mouse IgM (HRP) was purchased from Bioss, Beijing, China,. ECL kits were purchased from Biorigin, Beijing, China; RT Master Mix for qPCR II(MCE, State of New Jersey, USA); SYBR Green qPCR Master Mix (Low ROX) (MCE, State of New Jersey, USA).

#### Instruments

AB-SCIEX API 6500 QTRAP MS-WORKSTATION (MS BENCH) MOBILE LAB BENCH(Applied Biosystems, Foster City,USA); QuantStudio^™^ 6 Flex Real-Time PCR System, 384 wells(Applied Biosystems, Foster City,USA); Mini-PROTEAN^®^ Tetra Cell, Mini Trans-Blot^®^ Module, and PowerPac^™^ Basic Power Supply(Bio-Rad, State of California, USA); Pannoramic SCAN(3DHISTECH CaseViewer, Budapest, Hungary.

### Quality profiling for the main components of BGXJW by UPLC-MS/MS

0.1 g BGXJW powder was accurately weighed and added to 50 mL of methanol. The sample was vortexed for 1 min and sonicated for 30 min to make the stock solution with a concentration of 2 mg/mL. Take an appropriate amount of the prepared stock solution and dilute it by 1, 10, and 100 folds with ultrapure water to form sample solutions for use respectively. Internal standard solution (500 ng/mL buspirone hydrochloride and 500 μg/mL phenytoin sodium): Accurately weigh an appropriate amount of buspirone hydrochloride (TRC, Toronto, Canada) and phenytoin sodium (Bidepharm, Shanghai, China) control samples, dissolve them in DMSO and prepare a stock solution with buspirone hydrochloride and phenytoin sodium concentrations of 1.00 mg/mL respectively. Accurately measure an appropriate amount of the stock solution and dilute it with water until the concentration of buspirone hydrochloride is 500 ng/mL and the concentration of phenytoin sodium is 500 μg/mL of mixed internal standard solution.100 µL of each sample solution was added to 100 µL of mixed internal standard solution and vortexed for 1 min, then centrifuged at 14,000 rpm for 10 min, respectively. The supernatants were taken for injection analysis. The components of BGXJW were analyzed by AB-SCIEX API 6500 QTRAP MS-WORKSTATION (MS BENCH) MOBILE LAB BENCH. UPLC-MS/MS detection of liquid chromatographic conditions is shown in Additional file 1: Table S1.

### Animal experiments

Male C57BL/6 mice (SPF, SCXK [J] 2019-0010), 8 weeks old, were purchased from SPF Biotechnology Co., Ltd (Beijing, China). Mice were maintained in a controlled environment (23 ± 1 °C, lights on from 6:00 a.m. to 6:00 p.m.) with free access to water and food. All animal procedures were approved by the Institutional Animal Care and Use Committee at the Capital Medical University, Beijing, China. Approval number for ethical (IACUC-2021-0008).

To test the acute toxicity of BGXJW, mice were randomly divided into five groups: control (ctrl) group, BGXJW-treated (134, 268, 536, and 1072 mg/kg) groups. Each group has 6–10 mice. All mice were fed a control Lieber-DeCarli liquid diet for 15 days. Starting from the 5th day, mice in BGXJW-treated groups were further fed with BGXJW at 134, 268, 536 and 1072 mg/kg by oral gavage once a day for the following ten days, while mice in the ctrl group were given 0.9% saline at equal volumes daily. On the last day, all mice were sacrificed nine hours past gavage. The serum, liver, kidney, and small intestine were collected for subsequent experiments.

To exam if BGXJW could protect mice from alcohol-induced liver injury, the acute-on-chronic NIAAA model of ALD was chosen. The mice were randomly divided into five groups (6–10 mice per group): control (ctrl), ethanol (EtOH), and EtOH + BGXJW (33, 67, and 134 mg/kg) groups. All mice were fed the control Lieber-DeCarli liquid diet for the first five days to adapt liquid diet, then mice in EtOH + BGXJW groups were fed Lieber-DeCarli liquid diet containing 5%(v/v) ethanol for the next 10 days, meanwhile, BGXJW were given at indicated doses by oral gavage once a day. Mice in the ctrl group were fed a control Lieber-DeCarli liquid diet and equal volumes of 0.9% saline daily during these 10 days, while mice in the EtOH group were fed Lieber-DeCarli liquid diet containing 5%(v/v) ethanol and equal volumes of 0.9% saline daily. On the last day, all mice were administrated corresponding doses of BGXJW or equal volumes of saline. One hour later, the ctrl group was administrated with 45%(wt/v) maltodextrin solution (9 g/kg), EtOH group and EtOH + BGXJW groups were administrated with 31.5%(v/v) ethanol. All mice were sacrificed, and the serum and liver were collected for subsequent experiments after another nine hours.

To explore if BGXJW could relieve drunkenness, the alcohol binge-drinking model of ALD was used. The mice were divided into ctrl, EtOH, and EtOH + BGXJW groups. The detailed procedure could be seen in Fig. 7A. After fasting for 12 h, mice in the EtOH + BGXJW group were given 134 mg/kg BGXJW while mice in the ctrl and EtOH groups were given 0.9% saline for the corresponding volume. 30 min later, the ctrl group was given 0.9% saline, EtOH group and EtOH + BGXJW group were given 3 g/kg ethanol for the same volume. This operation was repeated 20 min later. Subsequently, the drunkenness situation of mice was detected and being recorded. The plasma samples at indicated time points were collected, centrifugated and immediately tested by gas chromatography.

### Biochemical test

The serum samples were collected after centrifuging at 3000 rpm, 4 °C for 15 min and stored at − 80 °C. Serum alanine aminotransferase (ALT), aspartate aminotransferase (AST), triglyceride (TG), creatine kinase MB (CK-MB), creatine kinase MM(CK-MM) concentrations after indicated treatments were determined by the related kits according to the manufacturer’s instructions. The ALT, AST, TG, CK-MB and CK-MM kits were purchased from Nanjing Jiancheng Bioengineering Institute, NanJing, China.

50 mg of liver tissue was homogenized in 500 µL ethanol absolute, then centrifuged at 2500 rpm for 10 min. Triglyceride (TG), total cholesterol(T-CHO)concentrations after indicated treatments were determined by the related kits according to the manufacturer’s instructions.The TG, T-CHO kits were purchased from Nanjing Jiancheng Bioengineering Institute, NanJing, China.Hepatic triglyceride and total cholesterol levels was normalized to tissue wet weight and expressed as mg/g of liver.

### ELISA assay

Frozen liver tissues were homogenized with RIPA Lysis Buffer (Biorigin, Beijing, China). TNF-α, IL-6 and IL-10 in liver lysates were determined by TNF-α, IL-6, and IL-10 ELISA kits according to the instructions. These ELISA kits were purchased from CLOUD-CLONE CORP. Wuhan, China.

### Western blot

Take part of frozen liver tissues and homogenize with RIPA Lysis Buffer (Biorigin, Beijing, China). Equal amounts of protein were separated using 8–12% sodium dodecyl sulfate–polyacrylamide-gel electrophoresis and transferred onto a polyvinylidene fluoride membrane (MILLIPORE, Massachusetts, USA). The membranes were blocked with 5% skim milk for 1 h and then incubated with indicated primary antibodies overnight at 4 °C, followed by a horseradish peroxidase-conjugated secondary antibody. The antibodies used against CYP2E1, SCD1, FASN, and PPAR-α were purchased from Abcam (Massachusetts, USA); antibodies against ADH1 and ALDH2 were purchased from Boster (California, USA) and Cell Signaling Technology (Boston, USA) separately. Antibodies against SREBP-1 (A-4) were purchased from Santa Cruz Biotechnology (Santa Cruz, USA). Antibodies against β-Actin were purchased from Bioworld (Minnesota, USA). HRP-Goat Anti-Rabbit IgG (H + L) was purchased from Biorigin (Beijing, China). Goat Anti-Mouse IgM (HRP) was purchased from Bioss (Beijing, China). ECL kits were purchased from Biorigin, Beijing, China.

### RT-PCR

For RT-PCR analysis, total RNA from liver tissues was isolated with FastPure Cell/Tissue Total RNA Isolation Kit (Vazyme, Nanjing, China) according to the instruction, and subsequently were reverse-transcribed to cDNA using RT Master Mix for qPCR II(MCE, State of New Jersey, USA). RT-PCR was carried out on Applied Biosystems^®^ QuantStudio^™^ 6 Flex Real-Time PCR System, 384 wells using SYBR Green qPCR Master Mix (Low ROX) (MCE, State of New Jersey, USA). Target gene expression was calculated by the comparative CT method. All primers used are listed in Additional file 1: Table S5.

### Histology

Liver, kidney, and small intestine collected after indicated treatments were fixed with 4% paraformaldehyde for at least 24 h, and then the sections were dehydrated, paraffin-embedded, and subjected to standard hematoxylin and eosin staining, then imaged with light microscopy (3DHISTECH CaseViewer, Pannoramic SCAN).

Hepatic steatosis was stained by oil red O staining.

### Statistics

All data in experiments were expressed as mean ± standard error of the mean (SEM). The comparison between groups was evaluated by GraphPad Prism (GraphPad Software, San Diego, CA, United States). One-way analysis of variance or Tukey’s multiple comparison tests was used to perform statistical analyses. Statistically significant differences between groups were defined as *p* values no more than 0.05.

## Results

### BGXJW has no acute cardiac, hepatic, renal, or intestinal toxicities

**Fig. 1:**
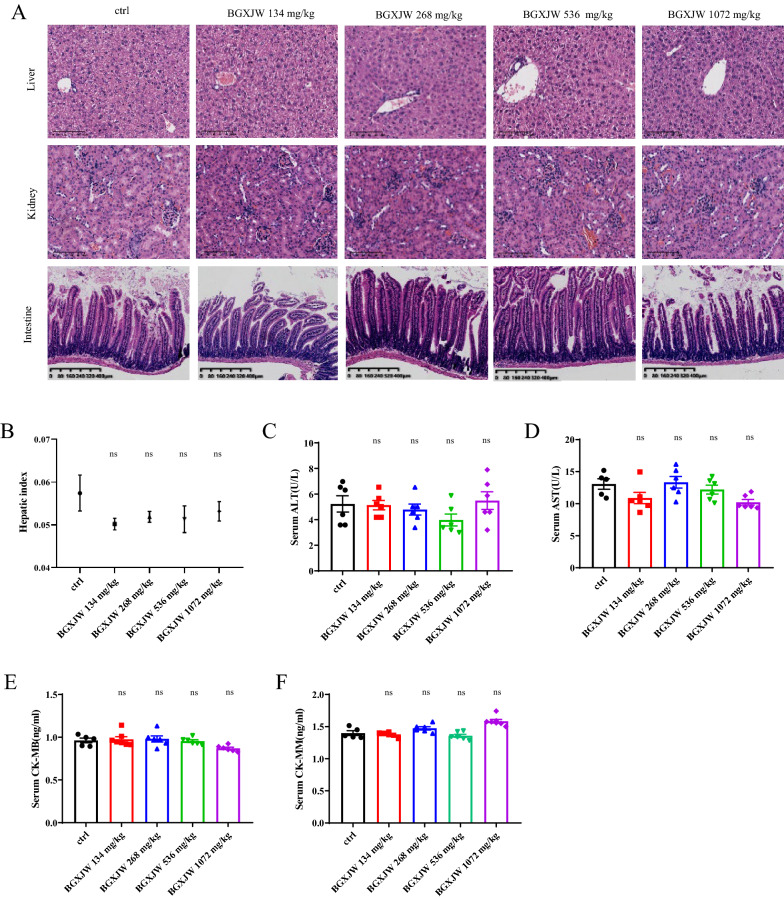
BGXJW has no obvious toxicity to liver, kidney, intestine and heart. C57BL/6 mice were fed with or without BGXJW for ten days. The tissue and serum samples were collected and tested. **A** Representative **H** and **E** staining of liver, kidney, intestine tissues. **B** Ratio of the liver to body weight. **C** ALT level in serum. **D** AST level in serum. **E** CK-MB level in serum. **F** CK-MM level in serum. All data are expressed as the mean ± SEM (n = 5). nsP > 0.05. *Crtl* contrl, *BGXJW* Bao-Gan-Xing-Jiu-Wan

To verify the safety of BGXJW, acute toxicity assays were performed. 2, 4, 8, and 16 times of the recommended clinical dose (9 g per day for adults) were converted into equivalent doses used in mice by the body surface area method and given to them. The histology examination of the liver, kidney, and intestine and the biochemical assays of the liver, heart, and kidney were conducted. As shown, the hepatic lobules are structurally intact, and the hepatocytes are radially arranged in strips centered on the blood vessels to form a clear hexagonal hepatic plate in both control and BGXJW (134, 268, 536, 1072 mg/kg)-treated groups (Fig. 1A). The structure of kidney and small intestine also show no significant difference between control and BGXJW-treated mice. The glomerular vascular collaterals of the kidney are thin and clear, and the surrounding tubules are in good condition in all groups. The small intestinal villi are with normal appearance, and the orderly organization of small intestinal mucosal cells, and smooth mucosal surface both in control and BGXJW-treated mice are observable (Fig. 1A). The results of CK-MM, CK-MB, ALT and AST, which are the serum biochemical indicators of heart and liver, also showed no significant differences between control and BGXJW (134, 268, 536, 1072 mg/kg)-treated mice (Fig. 1B–F). Collectively, these results demonstrate that BGXJW has no significant toxic effects on the structure and functions of the liver, heart, kidney, and intestinal.

### BGXJW effectively protects the liver from alcohol-induced liver injury

**Fig. 2:**
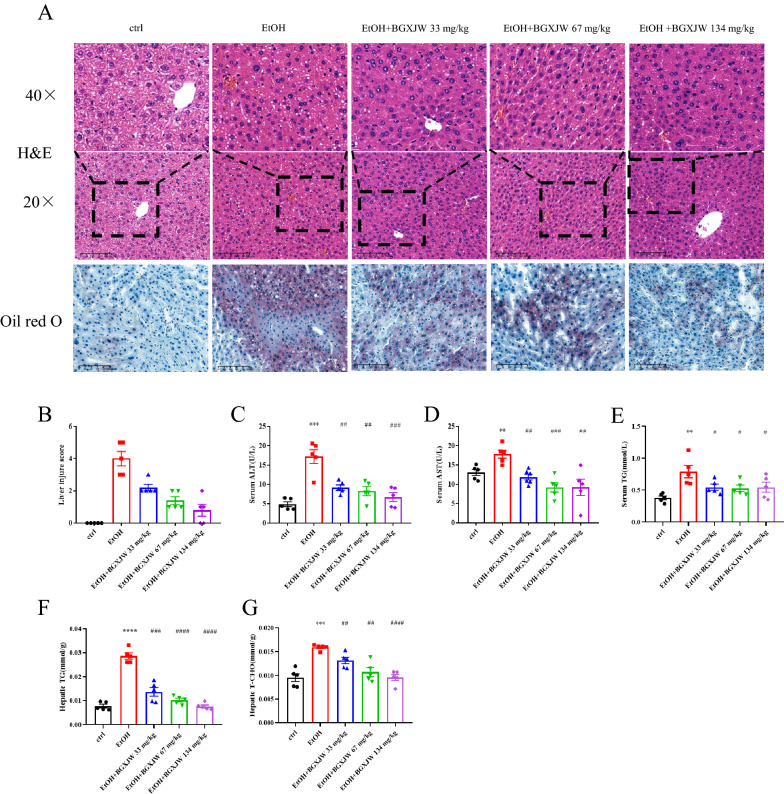
BGXJW alleviates ethanol-induced liver injury. C57BL/6 mice were grouped and fed as indicated in material and methods. The tissue and serum samples were collected and tested at the indicated time points. **A** Representative **H** and **E** staining and Oil Red O staining of liver tissues. **B** The relative liver injure score in each group. The degree of steatosis is graded as increase 1 point(mild, 5–33%), increase 2 points(moderate, 34–66%), and increase 3 points (severe, ≥ 67%), the degree of inflammatory infiltration is graded as increase 1 point (mild), increase 2 points (severe). **C** ALT level in serum. **D** AST level in serum. **E** Triglyceride (TG) level in serum. All data are expressed as the mean ± SEM (n = 5). **P < 0.01, ***P < 0.001, vs ctrl group; #P < 0.05, ##P < 0.01, ###P < 0.001, vs EtOH group. Crtl, contrl; EtOH,ethanol model; BGXJW, Bao-Gan-Xing-Jiu-Wan

As BGXJW has excellent outcomes on ALD clinically, we examined whether BGXJW could also protect against alcohol-induced liver injury in acute-on-chronic NIAAA mice. It was observed that ethanol exposure induced irregular arrangement of hepatocytes, lipid vesicles in the cytosolic compartment, and inflammatory infiltration in the liver, and these pathologic changes were relieved by BGXJW treatments (33, 67, 134 mg/kg) in a dose-dependent manner (Fig. 2A). The alleviation of alcohol-induced steatosis by BGXJW were also exhibited in the oil-red O staining results (Fig. 2A). In detail, the degree of liver damage in mice was scored based on the H&E staining results in each group, and the lesion score were 0.0 ± 0.0 (control group), 4.0 ± 1.0 (EtOH group), 2.2 ± 0.4 (EtOH + BGXJW 33 mg/kg group), 1.4 ± 0.5 (EtOH + BGXJW 67 mg/kg group) and 0.8 ± 0.8(EtOH + BGXJW 134 mg/kg group) respectively (Fig. 2B). Liver lesion induced by alcohol were half relieved by BGXJW at the dose of 33 mg/kg, and completely reversed by BGXJW at the dose of 134 mg/kg. Serum ALT, AST, and TG levels in mice were also examined (Fig. 2C–E). Interestingly, BGXJW exhibits a significant dose relationship in regulating TG and T-CHO levels in the liver (Fig. 2F–G). As shown, compared to the control mice, ALT, AST, TG and T-CHO levels increased significantly in the EtOH group, while these parameters decreased gradually with BGXJW administration at increasing doses. Collectively, these results show that BGXJW is effective in preventing alcoholic liver injury, hepatic lipid accumulation, and inflammatory cell accumulation.

Since BGXJW at all doses used above were effective in preventing alcoholic liver injury, and BGXJW at the dose of 134 mg/kg showed the most ideal result, we decided to adopt 134 mg/kg BGXJW in the following tests.

### BGXJW administrations regulate key proteins in lipid metabolism pathways modified by alcohol taken

As shown above, hepatic steatosis is the dominant pathogenetic feature of alcohol-induced liver injury and could be reversed by BGXJW treatment, we investigated the mechanisms of BGXJW on lipid metabolism disorder induced by alcohol from aspects of lipid synthesis and fatty acid β-oxidation.

**Fig. 3:**
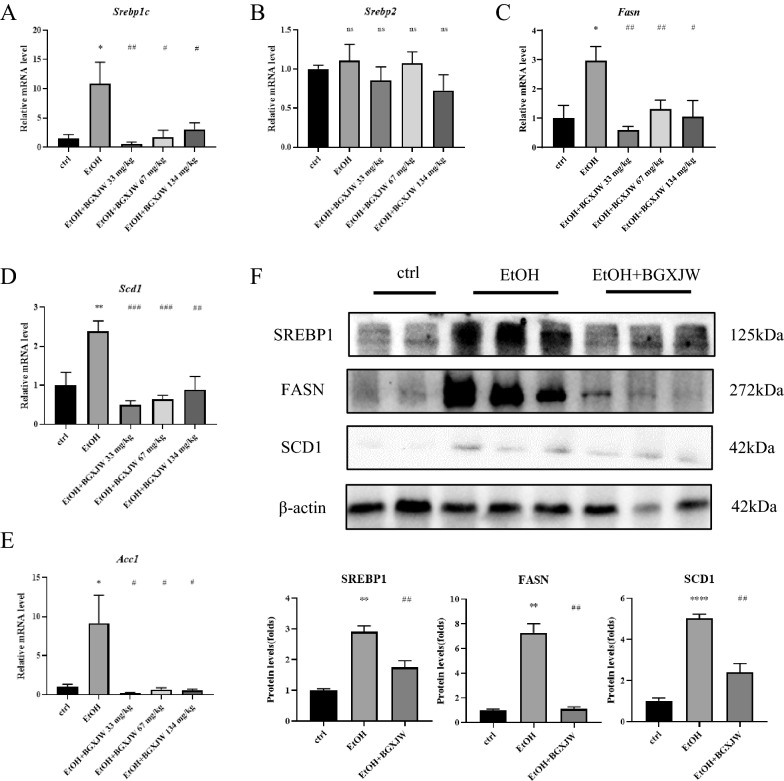
BGXJW administrations downregulate the mRNA and protein levels of key enzymes in the lipid synthesis pathway increased by alcohol. C57BL/6 mice were treated as indicated in material and methods. The mRNA and protein were collected and tested respectively. **A**–**E** The mRNA levels of SREBP1c, SREBP2, FASN, SCD1, ACC1 in liver tissues were subjected to RT-qPCR analysis. **F** SREBP1c, FAS, SCD1 in liver lysates with indicated treatment were determined by western blot. All data are expressed as the mean ± SEM (n = 3). *P < 0.05, **P < 0.01 vs ctrl group; nsP > 0.05, #P < 0.05, ##P < 0.01, ###P < 0.001 vs EtOH group. *Crtl* contrl, *EtOH*ethanol model, *BGXJW* Bao-Gan-Xing-Jiu-Wan

For lipid synthesis, we tested the expression of key lipid synthesis-associated genes in the NIAAA model mice treated with or without BGXJW. It was observed that alcohol taken significantly induced the mRNA transcription of lipid synthesis transcriptional regulator factor SREBP1c, also its downstream responsers FASN, SCD1 and ACC1, while BGXJW treatment restored them to normal levels (Fig. 3A–E). Alcohol and BGXJW both had no significant effect on the mRNA level of SREBP2 (Fig. 3B). The protein levels of SREBP1, FASN, and SCD1 were also detected (Fig. 3F). Compared with the ctrl group, alcohol consumption increased the protein levels of SREBP1, FASN, and SCD1, while BGXJW administration reversed it.

**Fig. 4:**
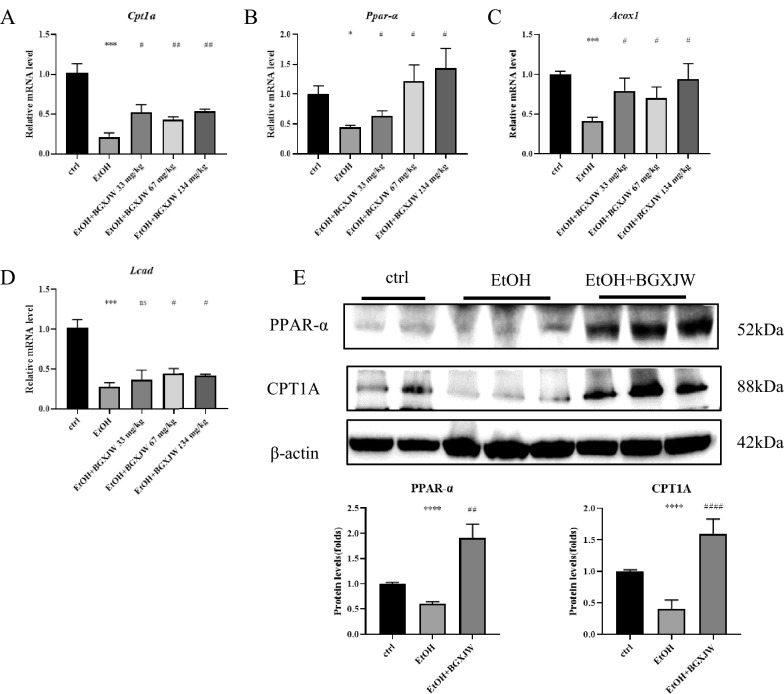
BGXJW administrations upregulate mRNA and protein levels of key enzymes of β-oxidation reduced by alcohol. C57BL/6 mice were grouped and fed as indicated in material and methods. The mRNA and protein were collected and tested at the indicated time points, respectively. **A**–**D** The mRNA levels of CPT1A, PPAR-α, ACOX1, LCAD, in liver tissues were subjected to RT-qPCR analysis. **E** PPARα, CPT1A in liver lysates were also determined by western blot. All data are expressed as the mean ± SEM (n = 3). *P < 0.05, **P < 0.01, ***P < 0.001 vs ctrl group; nsP > 0.05, #P < 0.05, ##P < 0.01, ###P < 0.001, ####P < 0.0001 vs EtOH group. Crtl, contrl; EtOH,ethanol model; BGXJW, Bao-Gan-Xing-Jiu-Wan

The key regulatory pathway of fatty acid β-oxidation was also investigated: carnitine palmitoyltransferase 1 (CPT1), the enzyme in the outer mitochondrial membrane that converts long-chain acyl-CoA species to their corresponding long-chain acyl-carnitines for transport into the mitochondria, also the rate-limiting step in long-chain fatty acid oxidation [52] peroxisome proliferator-activated receptor alpha (PPAR-α), a transcription factor which can regulate CPT1 to enhance fatty acid β oxidation; long-chain acyl-coenzyme A dehydrogenase (LCAD), the enzyme which catalyzes the initial step of the mitochondrial β-oxidation with different substrate-chain-length specificities; peroxisomal acyl-CoA oxidase 1 (Acox1), catalyzes the first and rate-limiting enzyme of the peroxisomal fatty acid β -oxidation pathway of very-long-chain fatty acids. Results showed that alcohol significantly reduced the mRNA level of PPAR-α, CPT1A, ACOX1, and LCAD, while BGXJW treatment reversed such a situation (Fig. 4A–D). A similar effect is also shown at the protein level. PPAR-α and CPT1A were downregulated by alcohol consumption and raised when receiving BGXJW treatment (Fig. 4E).

To sum up, it was shown that BGXJW could inhibit steatosis induced by alcohol taken by limiting lipid synthesis and rescuing fatty acid β-oxidation.

### BGXJW administrations inhibited inflammatory response induced by ethanol

**Fig. 5:**
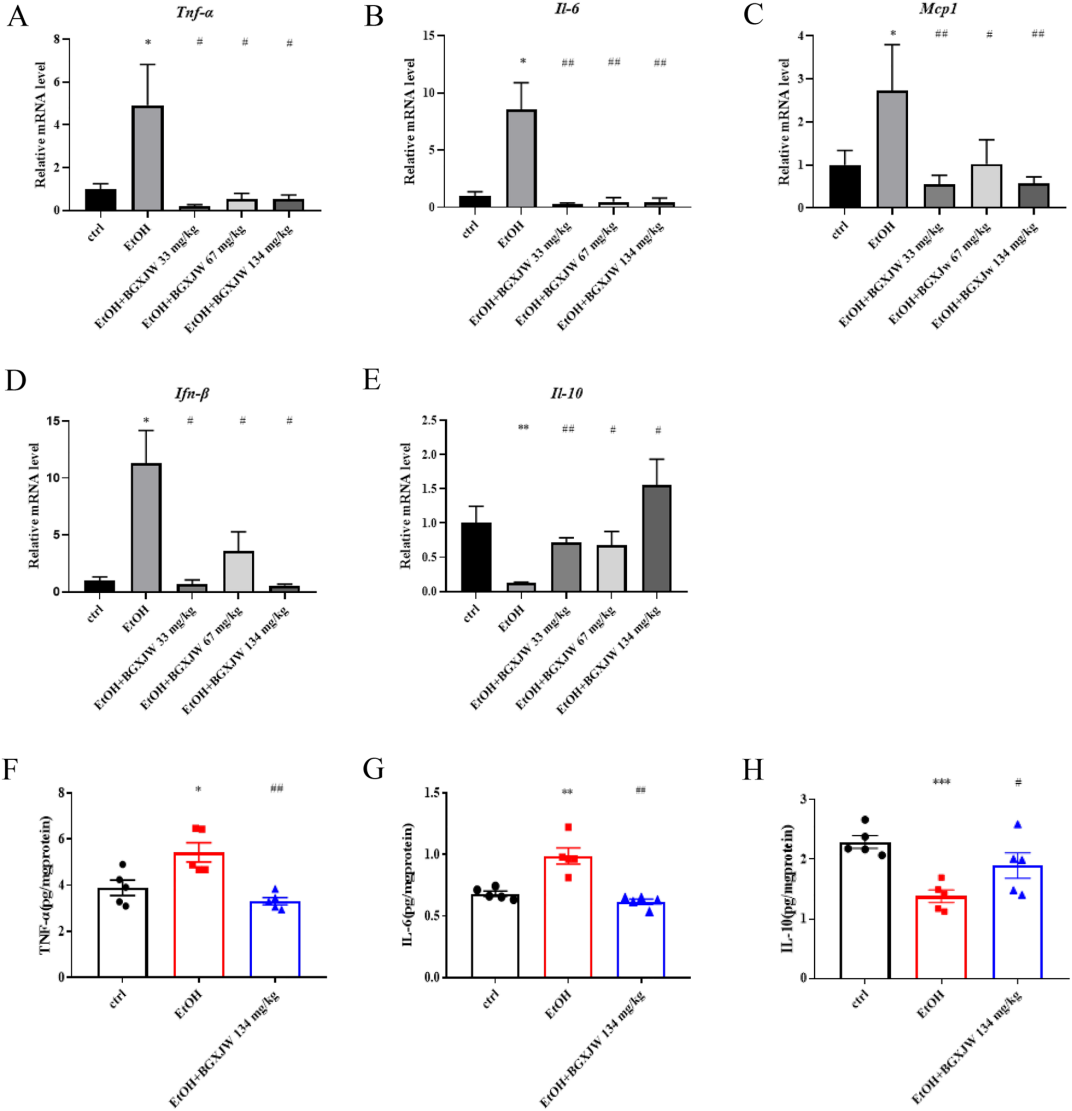
BGXJW administrations downregulate inflammatory response induced by alcohol. **A**–**E** The mRNA levels of TNF-α, IL-6, MCP1, IFN-β, IL-10 in liver tissues were subjected to RT-qPCR analysis. **B** TNF-α, IL-6, IL-10 in liver lysates with indicated treatment were determined by ELISA assay. All data are expressed as the mean ± SEM (n = 3). *P < 0.05, **P < 0.01, ***P < 0.001 vs ctrl group; nsP > 0.05, #P < 0.05, ##P < 0.01 vs EtOH group. *Crtl* contrl, *EtOH*ethanol model, *BGXJW* Bao-Gan-Xing-Jiu-Wan

Apart from steatosis, inflammatory infiltration in the liver induced by ethanol was also relieved by BGXJW treatment, the related mechanisms were further studied. The mRNA of pro-inflammatory and anti-inflammatory factors was examined. We found that alcohol taken upregulated pro-inflammatory cytokine TNF-α, IL-6, MCP-1, and IFN-β by ~ 3.8, 8.5, 2.7, and 11.3 folds separately, and downregulated the gene expression of anti-inflammatory IL-10 by ~ 8.1 folds. BGXJW treatment effectively reversed the upregulation of proinflammatory TNF-α, IL-6, MCP1, and IFN-β induced by alcohol, and recovered the anti-inflammatory IL-10 transcription (Fig. 5A–E). The ELISA results showed similar trends that compared to the ctrl group, alcohol consumption triggered hepatic TNF-α, IL-6 expression, and decreased IL-10 expression, while BGXJW administration reversed the effects. These results demonstrate that BGXJW could inhibit inflammatory responses induced by ethanol.

### BGXJW regulated alcohol metabolizing-associated enzymes

**Fig. 6:**
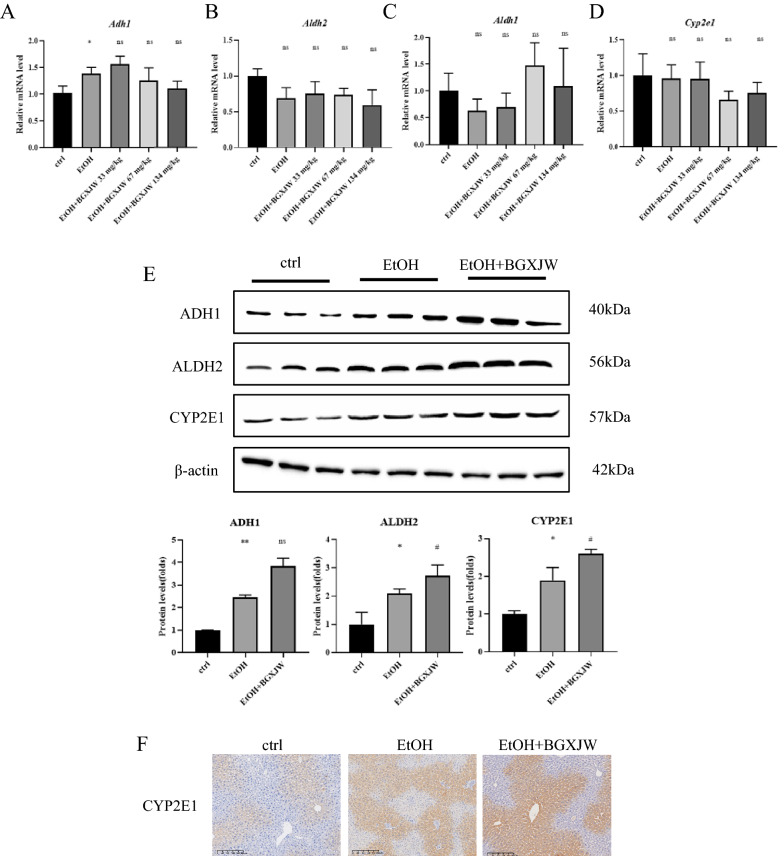
The metabolic enzymes ADH1, ALDH2 and CYP2E1 are significantly up-regulated by alcohol, and further up-regulated by BGXJW administration. **A**–**E** The mRNA levels of ADH1, ALDH2, ALDH1, CYP2E1 in liver tissues were subjected to RT-qPCR analysis. **E** Levels of ADH1, ALDH2, CYP2E1 in liver lysates after indicated treatment were determined by western blot, **F** Liver tissue sections were subjected to immunohistochemistry analysis of CYP2E1. All data are expressed as the mean ± SEM (n = 3). nsP > 0.05, *P < 0.05 vs ctrl group; nsP > 0.05 vs EtOH group. *Crtl* contrl, *EtOH* ethanol model, *BGXJW* Bao-Gan-Xing-Jiu-Wan

We also examined whether the alcohol metabolizing enzymes were regulated by BGXJW. As shown, no significant differences of Adh1, Aldh1, Aldh2, or Cyp2e1 at the mRNA level were observed among all groups (Fig. 6A–D). However, at the protein level, alcohol taken enhanced ADH1, ALDH2, and CYP2E1 quantity, and BGXJW administration further upregulated these three enzymes (Fig. 6E). The CYP2E1 was tested by immunohistochemical staining of the liver section in each group (Fig. 6G) and similar results were observed that BGXJW did not decrease, but elevated alcohol-induced CYP2E1 expression. It indicated that BGXJW might accelerate the metabolic efficiency of alcohol and decrease the accumulation of alcohol and acetaldehyde in the body to reduce alcohol-induced liver injury.

### BGXJW can reduce serum ethanol concentration and relieve drunkenness in acute alcohol-binge drinking mice model

**Fig. 7:**
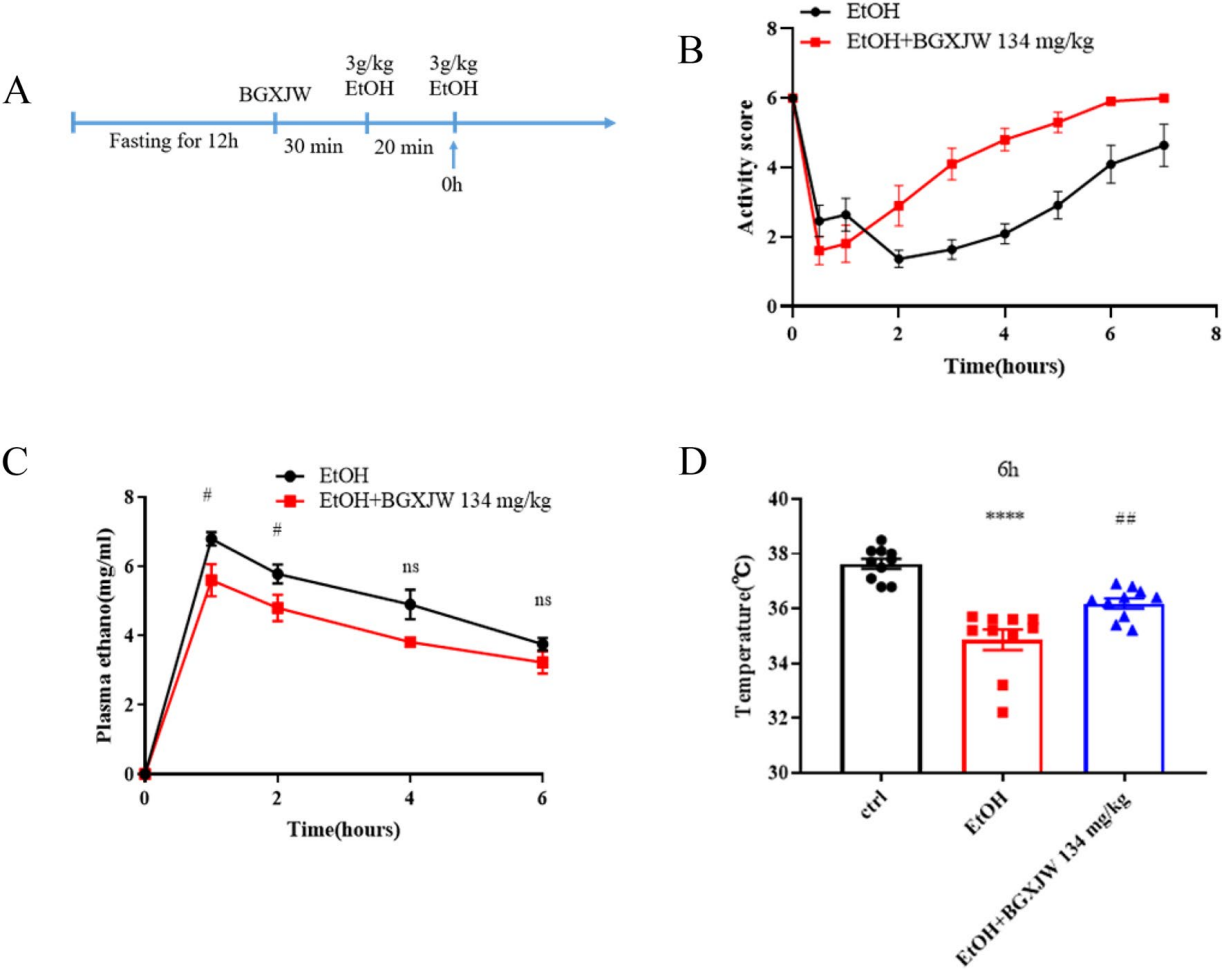
BGXJW administrations significantly reduce the plasma alcohol content, and effectively relieve the drunkenness of mice. **A** The schematic diagram of serum ethanol contents, activities body temperature test after acute ethanol taken below. C57BL/6 mice were given BGXJW or 0.9% saline by gavage. After 30 min, 3 g/kg ethanol was given twice by gavage with 20 min intervals. **B** The activity status of mice at each time point was scored after ethanol taken as in A. **C** Serum ethanol were subjected to gas chromatography. **D** The temperature of mice at 6 h after ethanol taken. All data are expressed as the mean ± SEM (n = 3). ****P < 0.0001 vs ctrl group; nsP > 0.05, #P < 0.05 vs EtOH group, ##P < 0.01 vs EtOH group. *Crtl* contrl, *EtOH* ethanol model, *BGXJW* Bao-Gan-Xing-Jiu-Wan

The above results present that BGXJW could promote alcohol metabolism enzyme expression, this suggests that BGXJW might promote alcohol metabolism and improve the drunkenness situation. So we investigated the serum ethanol concentration and the drunken situation in the alcohol-binge-drinking mice model. To simulate the human drinking mode, mice were given 3 g/kg alcohol twice with 20 min intervals post BGXJW or 0.9% saline treatment (Fig. 7A). After the last dose of alcohol, the duration of drunkenness and the activity ability recovery situation of mice were recorded. Data were exhibited in Tables 1, 2 and Fig. 7B. It could be seen that it would take 228.6 ± 42.6 min until the mice have righting reflection after alcohol taken, and 134 mg/kg BGXJW treatment could effectively reduce the duration to 132.5 ± 32.5 min. In addition, mice in the 134 mg/kg EtOH + BGXJW group could sober completely from drunkenness faster (312.0 ± 74.9 min) than that in the EtOH group (422.2 ± 48.5 min).

**Table 1:** Ethanol content in mouse plasma at various time points

Time (hours)	0	1	2	4	6
EtOH(mg/mL)	undetected	6.790 ± 0.339	5.779 ± 0.475	4.899 ± 0.610	3.74975 ± 0.316
EtOH + BGXJW 134 mg/kg (mg/ml)	undetected	5.595 ± 0.919	4.796 ± 0.761	3.910 ± 0.219	3.22 ± 0.554

**Table 2:** Mouse sober up schedule

	Recovery time of righting reflection (min)	Recovery time of normal mobility (min)
EtOH	228.6 ± 42.6	422.2 ± 48.5
EtOH + BGXJW 134 mg/kg	132.5 ± 32.5	312.0 ± 74.9

The detailed activities capacity at each timepoint after alcohol taken were also observed and scored according to the rating scale of mouse sobriety used previously (Table 3) [9, 20, 39], results showed that BGXJW could improve the drunkenness state and shorten the endurance after alcohol taken (Fig. 7B).

**Table 3:** Rating scale of mouse sobriety

Score	Definition
1	Loss of righting reflection
2	Recovery of righting reflection
3	Active swing of hind legs
4	Erect posture
5	The movement is normal, but obviously worse than normal
6	Normal mobility

The serum concentrations of ethanol were tested at 1 h, 2 h, 4 h and 6 h after alcohol administration by gas chromatography (Fig. 7C) as it could peak at 1 h post-alcohol treatment and then gradually decrease. BGXJW treatment did not alter the variation tendency of serum concentration of ethanol but effectively alleviated serum ethanol concentration at 1 h and 2 h. This is in accordance with the upper finding.

The body temperature of mice was measured at 6 h after the last dose of alcohol (Fig. 7D) the lower body temperature was observed in the EtOH group (34.9 ± 1.2 °C) compared to normal mice (37.6 ± 0.6 °C), while BGXJW treatment effectively alleviated the drop in body temperature caused by alcohol taken(36.2 ± 0.6 °C). Alcohol can have complex biphasic effects across time and dose,with psychomotor stimulation at relatively low doses, while high doses of alcohol can produce sedative effects [9]. In clinical studies, it has been found that human blood alcohol concentrations greater than 200 mg/dl are prone to hypothermia [53]. For mice with a dose of 3 g/kg of alcohol, their body temperature can be lower than that of normal mice, and it will further decrease as the alcohol dose increases [4, 23].

In summary, these results indicate that BGXJW can effectively relieve various drunk-induced symptoms.

### Characterization of BGXJW

BGXJW is a clinical experience-based Chinese herbal formula made from multiple herbal medicines. To character BGXJW, 18 elements were selected from the main components of each herb listed in the Chinese Pharmacopoeia and those reported in the literature. The contents of these 18 components in BGXJW were varied (Additional file 1: Table S2). The components with the highest contents in BGXJW were puerarin (14,700 μg/g), lithospermic acid B (4095 μg/g) and kakkalide (845 μg/g). Besides, the contents of chlorogenic acid (169 μg/g), 3,5-Dicaffeoylquinic acid (117 μg/g), cynaroside (114 μg/g), dihydromyricetin (100 μg/g) were all above 100 μg/g. The components, whose contents are no less than 10 mg/kg, were galangin (89.5 μg/g), tanshinone I (65.5 μg/g), tanshinone IIA (68 μg/g) and quercetin (32.5 μg/g).

## Discussion

Proper therapeutic management at the early stage of ALD may be helpful to prevent or ameliorate liver injury. BGXJW is often recommended for people to take before and after drinking alcoholic beverages as it can relieve their drunkenness and protect from ALD clinically. However, its scientific mechanism remains unknown. In the present research, we chose acute alcohol-binge drinking and the acute-on-chronic NIAAA models to uncover the effects and associated mechanisms of BGXJW in treating drunken and ALD mice. In addition, we also charactered BGXJW and investigated its toxicity. Our results demonstrate that BGXJW has no significant toxicities and can effectively alleviate drunkenness as well as prevent alcoholic liver injury. The mechanisms of our observation were mainly through regulating lipid metabolism, inhibiting inflammatory reactions and regulating alcohol metabolizing enzymes.

For the BGXJW safety assay, we set a series of high doses of BGXJW at 134, 268, 536 and 1072 mg/kg based on the clinical dose. Results show that BGXJW does not show acute cardio, hepatic, renal or intestinal toxicity either in histology or biochemistry assay (Fig. 1). We could conclude that BGXJW is safe even in the condition of overdose. As ≥ 134 mg/kg BGXJW had no significant toxicity in the liver, the single-dosing BGXJW-treated group was chosen for subsequent experiments. We tested if BGXJW could protect mice from alcohol-induced liver injury in the NIAAA mice model, and the results showed that BGXJW could prevent alcoholic liver injury such as steatosis in a dose-dependent manner (Fig. 2). Notably, the doses of BGXJW were 33, 67, and 134 mg/kg, corresponding to 0.5, 1, 2 times of that used clinically respectively. 33 mg/kg BGXJW could significantly alleviate lipid accumulation as the liver injury index score in this group was 0.55 times of that in the EtOH group. Besides, 134 mg/kg BGXJW could completely inhibit alcohol-induced liver injury (Fig. 2B). To sum up, these two results illustrate that BGXJW has a wide range of therapeutic doses, which is in accordance with its clinical application.

Subsequently, we explored the mechanism of BGXJW preventing alcoholic liver injury. As BGXJW can reduce alcoholic steatosis in the NIAAA model, we first focused on the effects of BGXJW in alcoholic-induced lipid metabolism disorder. The main cause of steatosis is the synthesis and deposition of fatty acids in hepatocytes, thus the lipid synthesis pathway was tested. Previous studies have shown that alcohol could increase the synthesis of liver lipids by enhancing FASN, ACC, SCD1 expression through the upregulation of transcription factor SREBP-1c [31, 35, 54, 55]. ACC facilitates the conversion of acetyl CoA into malonyl CoA by feeding it into the fatty acid de novo synthesis pathway [21]. Fatty acid synthase (FASN, also known as Fas), a key enzyme of fatty acid synthesis contributing to steatosis, catalyzes malonyl-CoA to palmitate [32]. SCD1 overexpression promotes the conversion of long-chain saturated fat to long-chain monounsaturated fat and promotes fat storage [1, 46] so mice with SCD1 deficiency have better insulin sensitivity and reduced liver fat [1]. Our results showed that alcohol taken enhanced FASN, ACC, and SCD1 expression at both mRNA and protein levels, which is consistent with the previous research, while BGXJW could significantly reverse the effects and inhibit lipid synthesis.

Fatty acid β-oxidation fuels hepatic ketogenesis, which provides a major source of energy once hepatic glycogen stores become depleted during prolonged fasting and periods of higher energy demands, it is also the main pathway of fatty acids consumption and degradation [27]. The effect of BGXJW on alcohol-induced fatty acids β-oxidation pathway dysregulation was further examined. We included the critical enzymes involved in fatty acids β-oxidation. It could be seen that CPT1 and PPAR-α were down-regulated in both mRNA and protein levels by alcohol administration, and BGXJW reversed the effects. PPAR-α has been reported to regulate CPT1 to enhance fatty acid β oxidation [18] and ethanol metabolite acetaldehyde accumulation downregulates PPAR-α to inhibit CPT1 function and decrease the fatty acid β oxidation [45]. In addition, PPAR-α activation affected the SREBP-1c/FASN pathway to alleviate ALD [26, 51]. Acox1, a rate-limiting enzyme of the peroxisomal fatty acid β -oxidation pathway of very-long-chain fatty acids, was also inhibited in mRNA levels after alcohol was taken and reversed by BGXJW in mRNA level [10]. LCAD, which catalyzes the dehydrogenation of long-chain fatty acid species with chain lengths of 12 to 22 carbon atoms [14] was also inhibited by alcohol taken, while BGXJW has no significant effect on its transcription. Collectively, BGXJW may regulate lipid metabolism against alcoholic liver injury by modifying the transcription and translation of proteins in the lipid synthesis and fatty acid β oxidation pathways.

During the experiments, we observed slight inflammatory cell infiltration in the liver of the NIAAA mice model, and it was effectively reduced after BGXJW intervention. Consistently, we examined the inflammatory response. It showed that BGXJW significantly abrogated the enhanced expression of proinflammatory TNF-α, IL-6 (Fig. 5F–G) and decreased expression of anti-inflammatory IL-10 both at the mRNA and protein level in the mice liver after ethanol taken (Fig. 5H), while BGXJW reverses the situation by decreasing the production of TNF-α, IL-6 and enhancing IL-10. BGXJW relieves ethanol-induced liver injury through the reduction of inflammation in the liver. To be noted, we also performed ELISA assay on the serum sample and no significant changes were detected between the ctrl group and the EtOH group (results not shown), it might be due to the inflammation in our NIAAA model being localized and limited in the liver. It has been reported that a combination of drugs containing pueraria can synergistically reduce the TNF-α, IL-6, and MCP1 induced by LPS-triggered TLR4-mediated NF-κB signaling pathway [15].

ADH1, CYP2E1, and ALDH2 are the key enzymes of alcohol metabolism, thus we also investigated the effect of BGJXW on these enzymes. The results implied that BGXJW increased the protein levels of ADH1, CYP2E1, and ALDH2 to accelerate the metabolism of alcohol. No regulation of alcohol or BGXJW on ADH1, ALDH2, or CYP2E1 was observed at the transcriptional level (Fig. 6A). It has been reported that alcohol upregulates ADH1, ALDH2, and CYP2E1 [3, 8, 25, 43] and altered expression of hepatic CYP2E1 by xenobiotic or physiological stimuli is largely mediated through post-transcriptional mechanisms that may include altered CYP2E1 mRNA translation and/or protein degradation [33]. This is in accordance with our observed results. Interestingly, some traditional Chinese medicines have been found to be effective through suppressing CYP2E1-mediated oxidative stress and enhancing the oxidant defense systems via the activation of Nrf2/HO-1pathway [7, 12]. Moreover, the facts that BGXJW up-regulates the protein levels of ADH1, CYP2E1, and ALDH2 may be related to the clinical observation that BGXJW could alleviate drunkenness and alcoholic liver injury, also imply that BGXJW might reduce blood alcohol concentration after alcohol intaken. Therefore, we chose the alcohol binge-drinking model to study the improvement of BGXJW on drunkenness and related performances. The results show that BGXJW could effectively reduce the concentration of ethanol in the blood, relieve drunkenness, and alleviate the hypothermia caused by central inhibition after drinking (Fig. 7B–D). A study found that Chinese medicine can also alleviate acute ALD by suppressing oxidative stress, inflammation, and apoptosis [11].

BGXJW is extracted from 9 herbs, to have a better understanding of its composition, the components were characterized. Puerarin (14,700 μg/g), of the highest contents in BGXJW, is the crucial component of roots and flos of *P. lobata*. Previous research showed that the extract of flos of *P. lobata* or puerarin can effectively prevent drunkenness in mice [60]. Puerarin alleviates alcoholic liver injury by down-regulating the expression of lipid synthesis enzyme, FASN, and pro-inflammatory factor, TNF-a and IL-1β [36, 61]. In chronic alcohol-fed mice, puerarin pretreatment significantly increased body weight and liver ADH activity in a dose-dependent manner [60]. Kakkalide (of 845 μg/g in BGXJW) is also a critical component in flos of *P. lobata* has been reported that can effectively reduce mortality and liver damage in mice with acute alcoholism [41]. In addition, kakkalide inhibited ROS-associated inflammation by inhibiting fatty acid β oxidation and IL-6 production [58]. Lithospermic acid B (of 4095 μg/g in BGXJW), tanshinone I (of 65.5 μg/g in BGXJW), tanshinone IIA (of 68 μg/g in BGXJW) are the components of roots of *S. miltiorrhiza*. Lithospermic acid B exerted anti-steatotic and anti-inflammatory effects against alcoholic liver injury by inducing SIRT1-mediated inhibition of CRP and ChREBP expression [59] and it could also alleviate acute ethanol-induced hepatocyte apoptosis through SIRT1-mediated deacetylation of p53 pathway [34]. Tanshinone I, dihydromyricetin, and chlorogenic acid have been reported to have certain preventive or therapeutic effects on alcoholic liver disease, nonalcoholic liver disease or inflammatory diseases [29, 42, 50]. Chlorogenic acid (of 169 μg/g in BGXJW) and dihydromyricetin (of 100 μg/g in BGXJW) protect liver function via altering lipid metabolism and enhancing alcohol metabolism, [5, 47]. All these reports indicated the synthetic action of these components would contribute to BGXJW’s therapeutic effect against ALD.

## Conclusion

Taken together, our experimental results suggest that BGXJW is safe, effective, and clinically worthy of generalization.

## Supplementary Information

**Additional file 1: Figure S1.** SIRT1 are significantly up-regulated by alcohol, and further up-regulated by BGXJW administration. Levels of SIRT1 in liver lysates after indicated treatment were determined by western blot (n=3). Crtl, contrl; EtOH,ethanol model; BGXJW, Bao-Gan-Xing-Jiu-Wan. **Figure S2.** TIC spectrum under positive and negative ion conditions (mixed). **Figure S3.** TIC spectrum under positive and negative ion conditions. **Table S1.** UPLC-MS/MS detection of liquid chromatographic conditions. **Table S2.** The Measured Concentrations of 18 Compounds in BGXJW. **Table S3.** Determination of Mass Spectrometry Parameters of 18 Compounds in BGXJW by UPLC-MS/MS. **Table S4.** Linear regression equation, correlation coefficient, linear range, detection limit and quantification limit of 18 compounds in BGXJW. **Table S5.** Primers.

## Data Availability

Data available on request from the authors.
