# Supplementary material for: A clinical experience-based Chinese herbal formula improves ethanol-induced drunken behavior and hepatic steatohepatitis in mice models
Source: Chin Med. 2023 May 1;18:47. doi: 10.1186/s13020-023-00753-5 (PMC10150545; doi:10.1186/s13020-023-00753-5)
Supplement: Supplementary file 1 — Additional file 1: Figure S1. SIRT1 are significantly up-regulated by alcohol, and further up-regulated by BGXJW administration. Levels of SIRT1 in liver lysates after indicated treatment were determined by western blot (n=3). Crtl, contrl; EtOH,ethanol model; BGXJW, Bao-Gan-Xing-Jiu-Wan. Figure S2. TIC spectrum under positive and negative ion conditions (mixed). Figure S3. TIC spectrum under positive and negative ion conditions. Table S1. UPLC-MS/MS detection of liquid chromatographic conditions. Table S2. The Measured Concentrations of 18 Compounds in BGXJW. Table S3. Determination of Mass Spectrometry Parameters of 18 Compounds in BGXJW by UPLC-MS/MS. Table S4. Linear regression equation, correlation coefficient, linear range, detection limit and quantification limit of 18 compounds in BGXJW. Table S5. Primers. [file 13020_2023_753_MOESM1_ESM.ppt]

## Slide 1
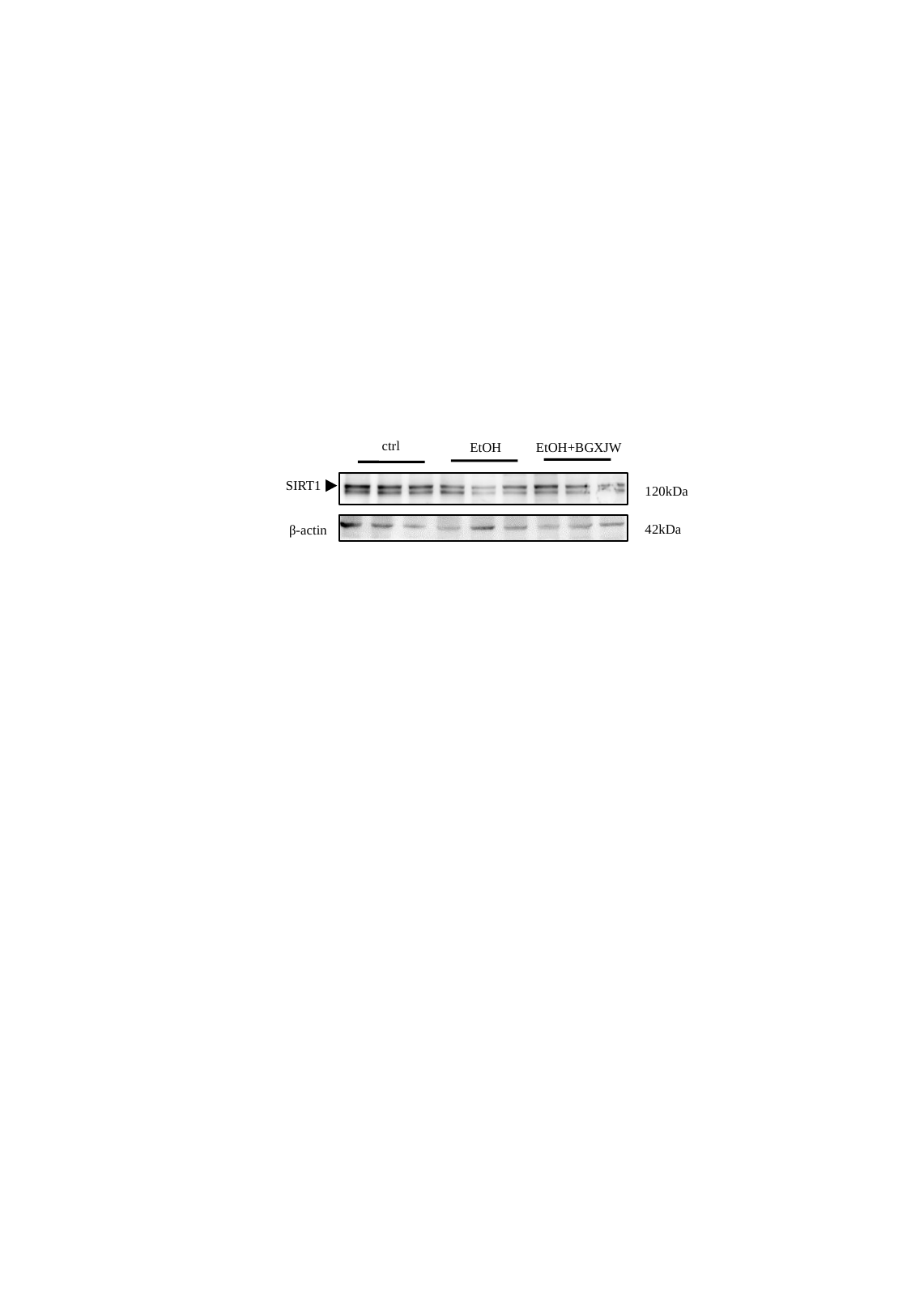

ctrl
EtOH
EtOH+BGXJW
SIRT1
β-actin
120kDa
42kDa

## Slide 2
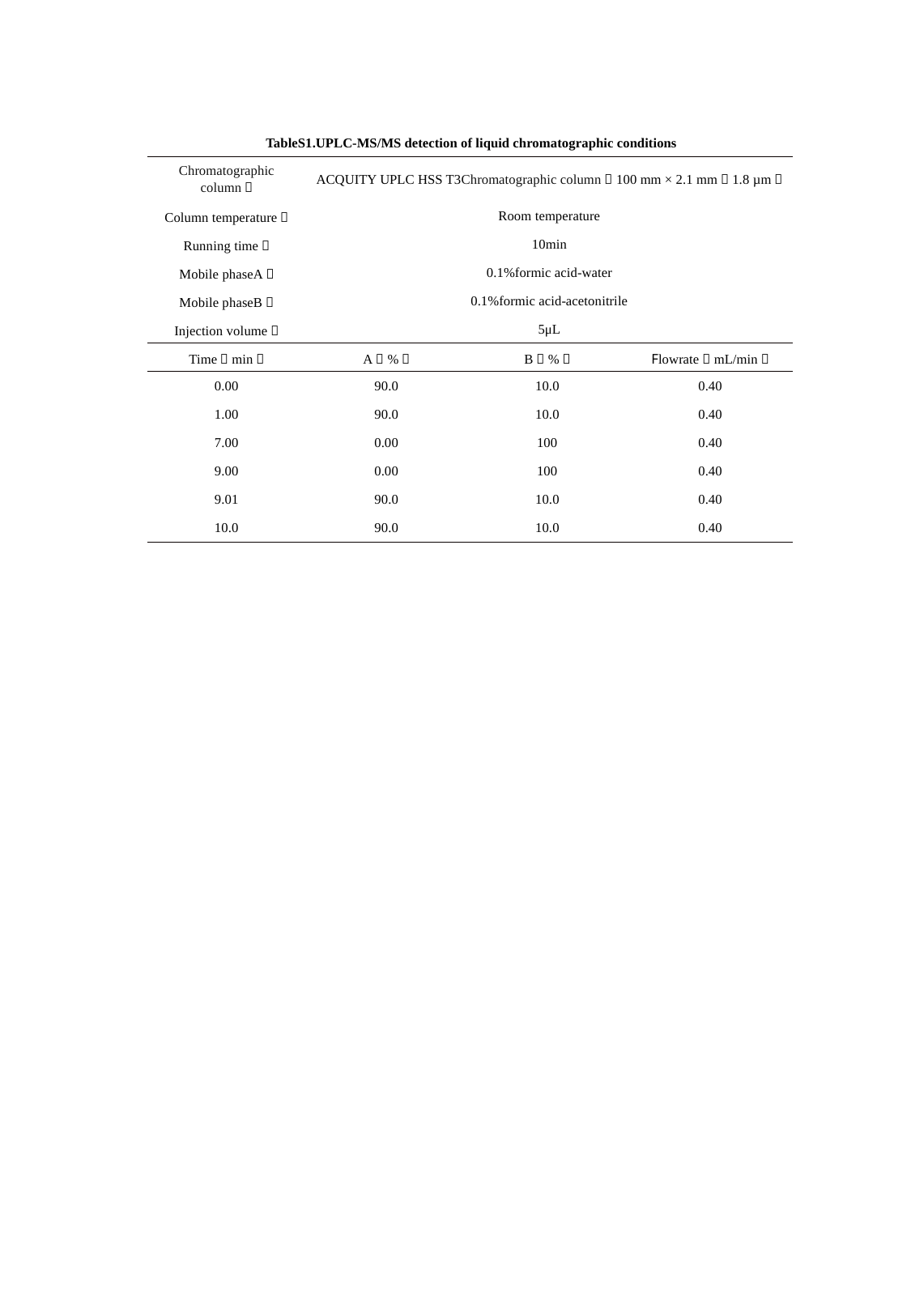

TableS1.UPLC-MS/MS detection of liquid chromatographic conditions
| Chromatographic column： | ACQUITY UPLC HSS T3Chromatographic column（100 mm × 2.1 mm，1.8 µm） | | |
| --- | --- | --- | --- |
| Column temperature： | Room temperature | | |
| Running time： | 10min | | |
| Mobile phaseA： | 0.1%formic acid-water | | |
| Mobile phaseB： | 0.1%formic acid-acetonitrile | | |
| Injection volume： | 5μL | | |
| Time（min） | A（%） | B（%） | Flowrate（mL/min） |
| 0.00 | 90.0 | 10.0 | 0.40 |
| 1.00 | 90.0 | 10.0 | 0.40 |
| 7.00 | 0.00 | 100 | 0.40 |
| 9.00 | 0.00 | 100 | 0.40 |
| 9.01 | 90.0 | 10.0 | 0.40 |
| 10.0 | 90.0 | 10.0 | 0.40 |

## Slide 3
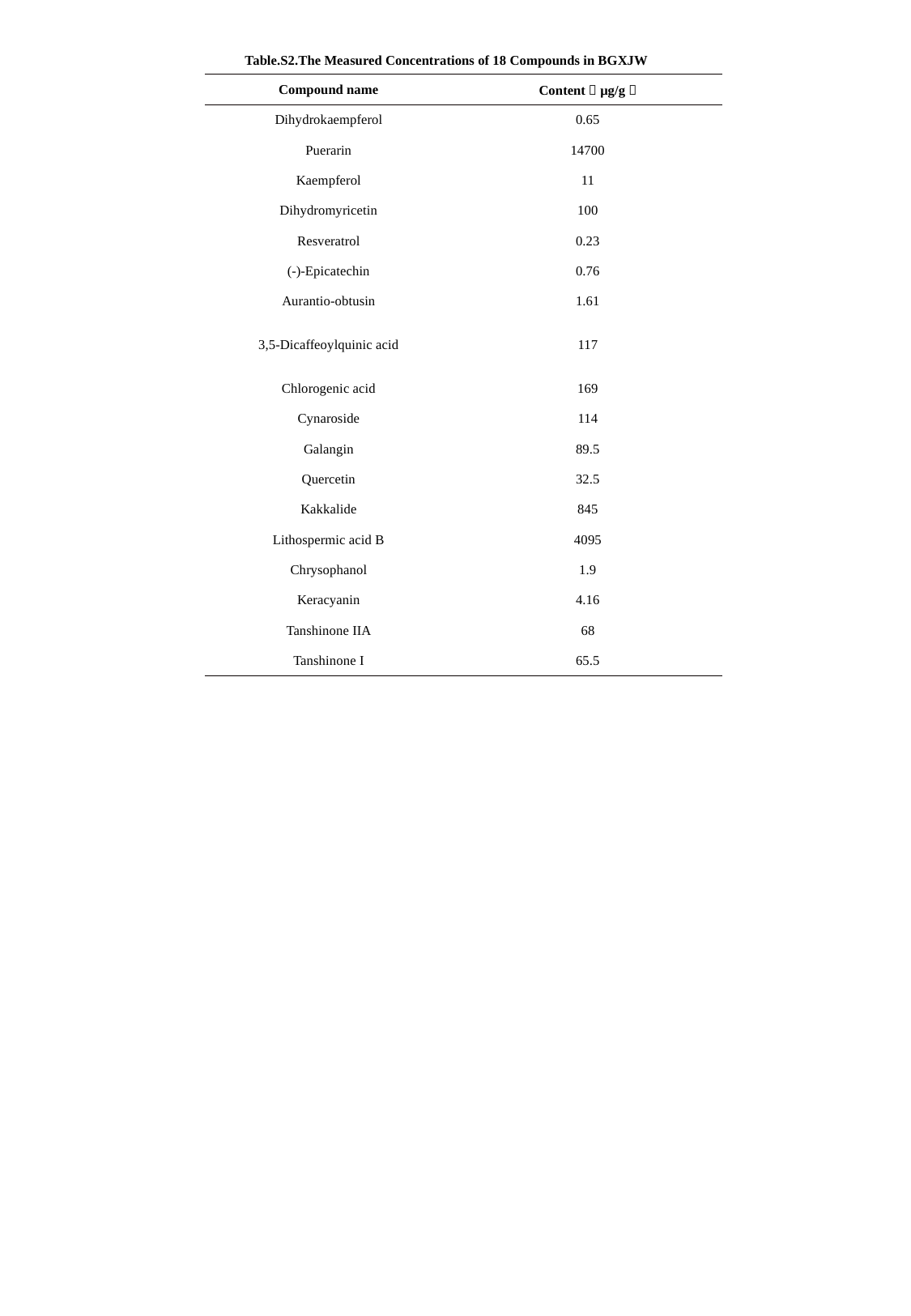

Table.S2.The Measured Concentrations of 18 Compounds in BGXJW
| Compound name | Content（μg/g） |
| --- | --- |
| Dihydrokaempferol | 0.65 |
| Puerarin | 14700 |
| Kaempferol | 11 |
| Dihydromyricetin | 100 |
| Resveratrol | 0.23 |
| (-)-Epicatechin | 0.76 |
| Aurantio-obtusin | 1.61 |
| 3,5-Dicaffeoylquinic acid | 117 |
| Chlorogenic acid | 169 |
| Cynaroside | 114 |
| Galangin | 89.5 |
| Quercetin | 32.5 |
| Kakkalide | 845 |
| Lithospermic acid B | 4095 |
| Chrysophanol | 1.9 |
| Keracyanin | 4.16 |
| Tanshinone IIA | 68 |
| Tanshinone I | 65.5 |

## Slide 4
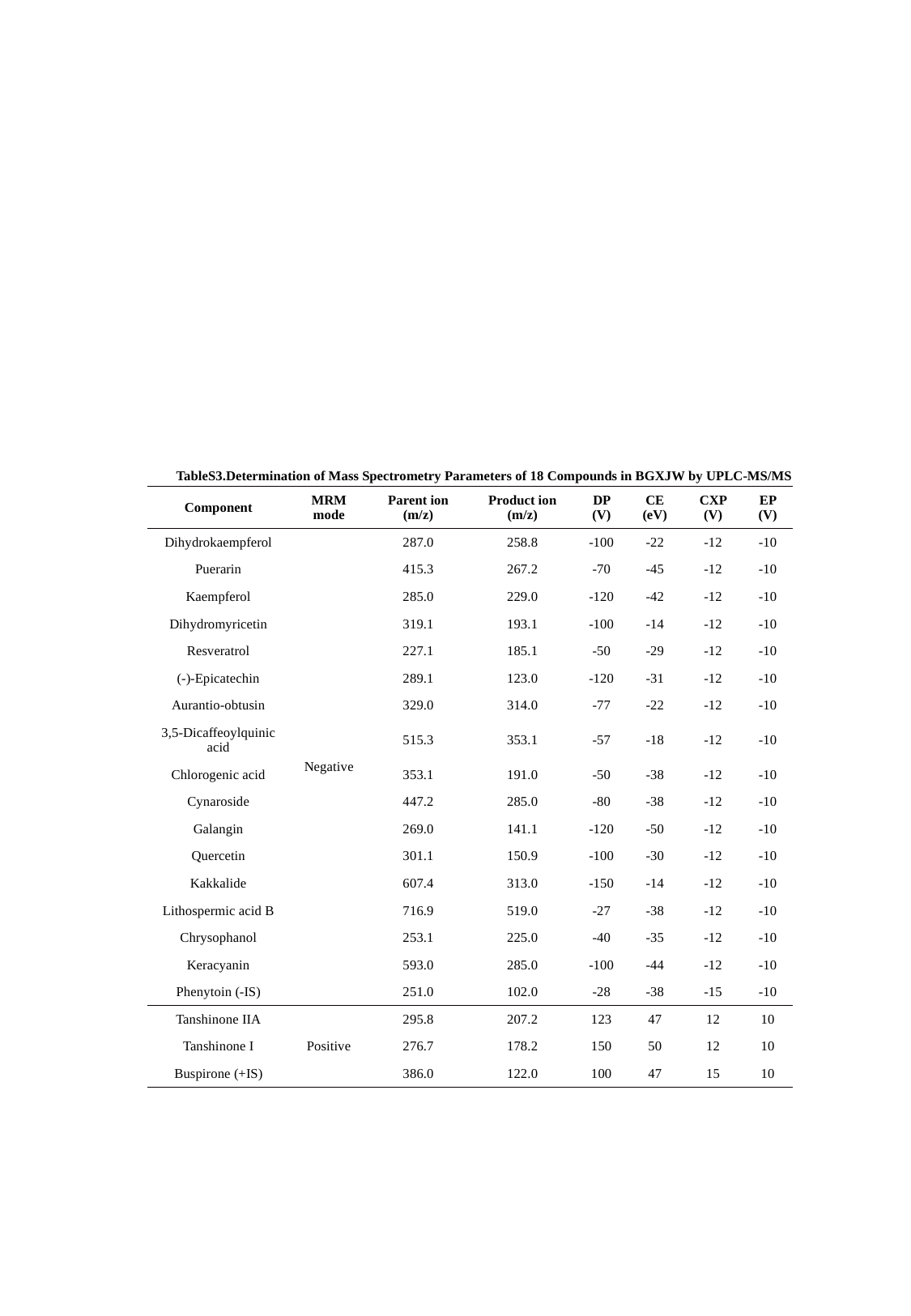

TableS3.Determination of Mass Spectrometry Parameters of 18 Compounds in BGXJW by UPLC-MS/MS
| Component | MRM mode | Parent ion (m/z) | Product ion (m/z) | DP (V) | CE (eV) | CXP (V) | EP (V) |
| --- | --- | --- | --- | --- | --- | --- | --- |
| Dihydrokaempferol | Negative | 287.0 | 258.8 | -100 | -22 | -12 | -10 |
| Puerarin | | 415.3 | 267.2 | -70 | -45 | -12 | -10 |
| Kaempferol | | 285.0 | 229.0 | -120 | -42 | -12 | -10 |
| Dihydromyricetin | | 319.1 | 193.1 | -100 | -14 | -12 | -10 |
| Resveratrol | | 227.1 | 185.1 | -50 | -29 | -12 | -10 |
| (-)-Epicatechin | | 289.1 | 123.0 | -120 | -31 | -12 | -10 |
| Aurantio-obtusin | | 329.0 | 314.0 | -77 | -22 | -12 | -10 |
| 3,5-Dicaffeoylquinic acid | | 515.3 | 353.1 | -57 | -18 | -12 | -10 |
| Chlorogenic acid | | 353.1 | 191.0 | -50 | -38 | -12 | -10 |
| Cynaroside | | 447.2 | 285.0 | -80 | -38 | -12 | -10 |
| Galangin | | 269.0 | 141.1 | -120 | -50 | -12 | -10 |
| Quercetin | | 301.1 | 150.9 | -100 | -30 | -12 | -10 |
| Kakkalide | | 607.4 | 313.0 | -150 | -14 | -12 | -10 |
| Lithospermic acid B | | 716.9 | 519.0 | -27 | -38 | -12 | -10 |
| Chrysophanol | | 253.1 | 225.0 | -40 | -35 | -12 | -10 |
| Keracyanin | | 593.0 | 285.0 | -100 | -44 | -12 | -10 |
| Phenytoin (-IS) | | 251.0 | 102.0 | -28 | -38 | -15 | -10 |
| Tanshinone IIA | Positive | 295.8 | 207.2 | 123 | 47 | 12 | 10 |
| Tanshinone I | | 276.7 | 178.2 | 150 | 50 | 12 | 10 |
| Buspirone (+IS) | | 386.0 | 122.0 | 100 | 47 | 15 | 10 |

## Slide 5
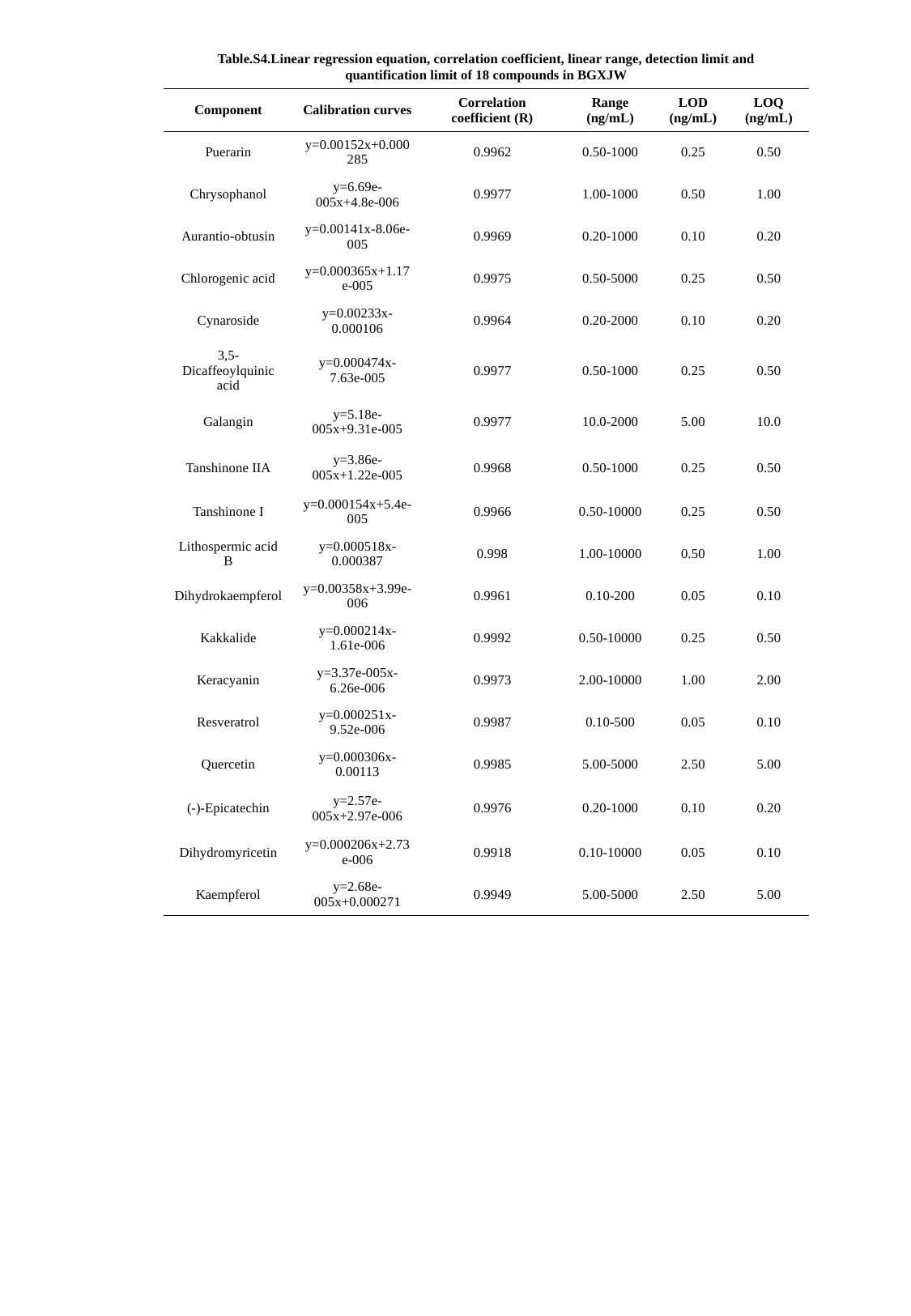

Table.S4.Linear regression equation, correlation coefficient, linear range, detection limit and quantification limit of 18 compounds in BGXJW
| Component | Calibration curves | Correlation coefficient (R) | Range (ng/mL) | LOD (ng/mL) | LOQ (ng/mL) |
| --- | --- | --- | --- | --- | --- |
| Puerarin | y=0.00152x+0.000285 | 0.9962 | 0.50-1000 | 0.25 | 0.50 |
| Chrysophanol | y=6.69e-005x+4.8e-006 | 0.9977 | 1.00-1000 | 0.50 | 1.00 |
| Aurantio-obtusin | y=0.00141x-8.06e-005 | 0.9969 | 0.20-1000 | 0.10 | 0.20 |
| Chlorogenic acid | y=0.000365x+1.17e-005 | 0.9975 | 0.50-5000 | 0.25 | 0.50 |
| Cynaroside | y=0.00233x-0.000106 | 0.9964 | 0.20-2000 | 0.10 | 0.20 |
| 3,5-Dicaffeoylquinic acid | y=0.000474x-7.63e-005 | 0.9977 | 0.50-1000 | 0.25 | 0.50 |
| Galangin | y=5.18e-005x+9.31e-005 | 0.9977 | 10.0-2000 | 5.00 | 10.0 |
| Tanshinone IIA | y=3.86e-005x+1.22e-005 | 0.9968 | 0.50-1000 | 0.25 | 0.50 |
| Tanshinone I | y=0.000154x+5.4e-005 | 0.9966 | 0.50-10000 | 0.25 | 0.50 |
| Lithospermic acid B | y=0.000518x-0.000387 | 0.998 | 1.00-10000 | 0.50 | 1.00 |
| Dihydrokaempferol | y=0.00358x+3.99e-006 | 0.9961 | 0.10-200 | 0.05 | 0.10 |
| Kakkalide | y=0.000214x-1.61e-006 | 0.9992 | 0.50-10000 | 0.25 | 0.50 |
| Keracyanin | y=3.37e-005x-6.26e-006 | 0.9973 | 2.00-10000 | 1.00 | 2.00 |
| Resveratrol | y=0.000251x-9.52e-006 | 0.9987 | 0.10-500 | 0.05 | 0.10 |
| Quercetin | y=0.000306x-0.00113 | 0.9985 | 5.00-5000 | 2.50 | 5.00 |
| (-)-Epicatechin | y=2.57e-005x+2.97e-006 | 0.9976 | 0.20-1000 | 0.10 | 0.20 |
| Dihydromyricetin | y=0.000206x+2.73e-006 | 0.9918 | 0.10-10000 | 0.05 | 0.10 |
| Kaempferol | y=2.68e-005x+0.000271 | 0.9949 | 5.00-5000 | 2.50 | 5.00 |

## Slide 6
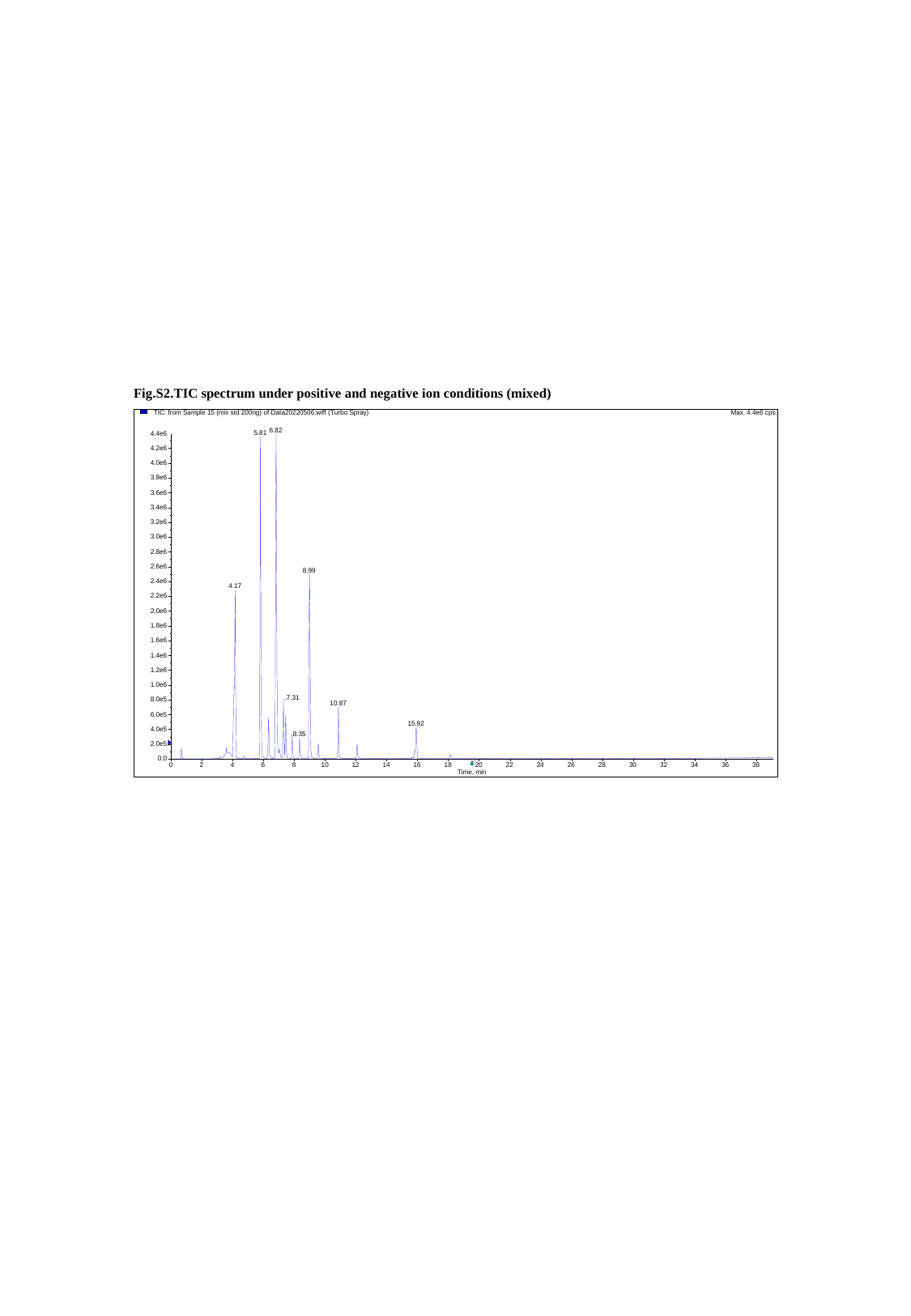

Fig.S2.TIC spectrum under positive and negative ion conditions (mixed)

## Slide 7
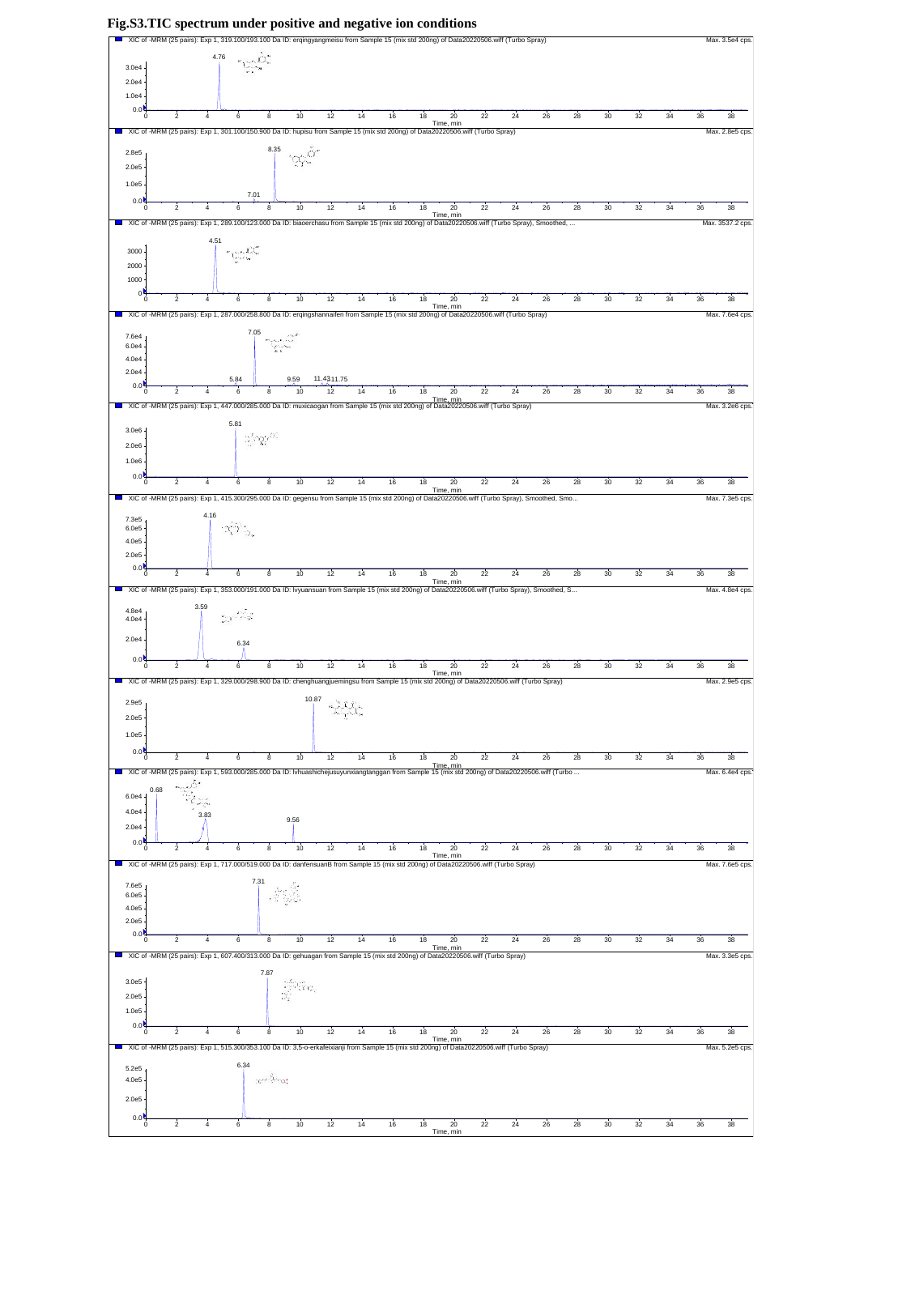

Fig.S3.TIC spectrum under positive and negative ion conditions

## Slide 8
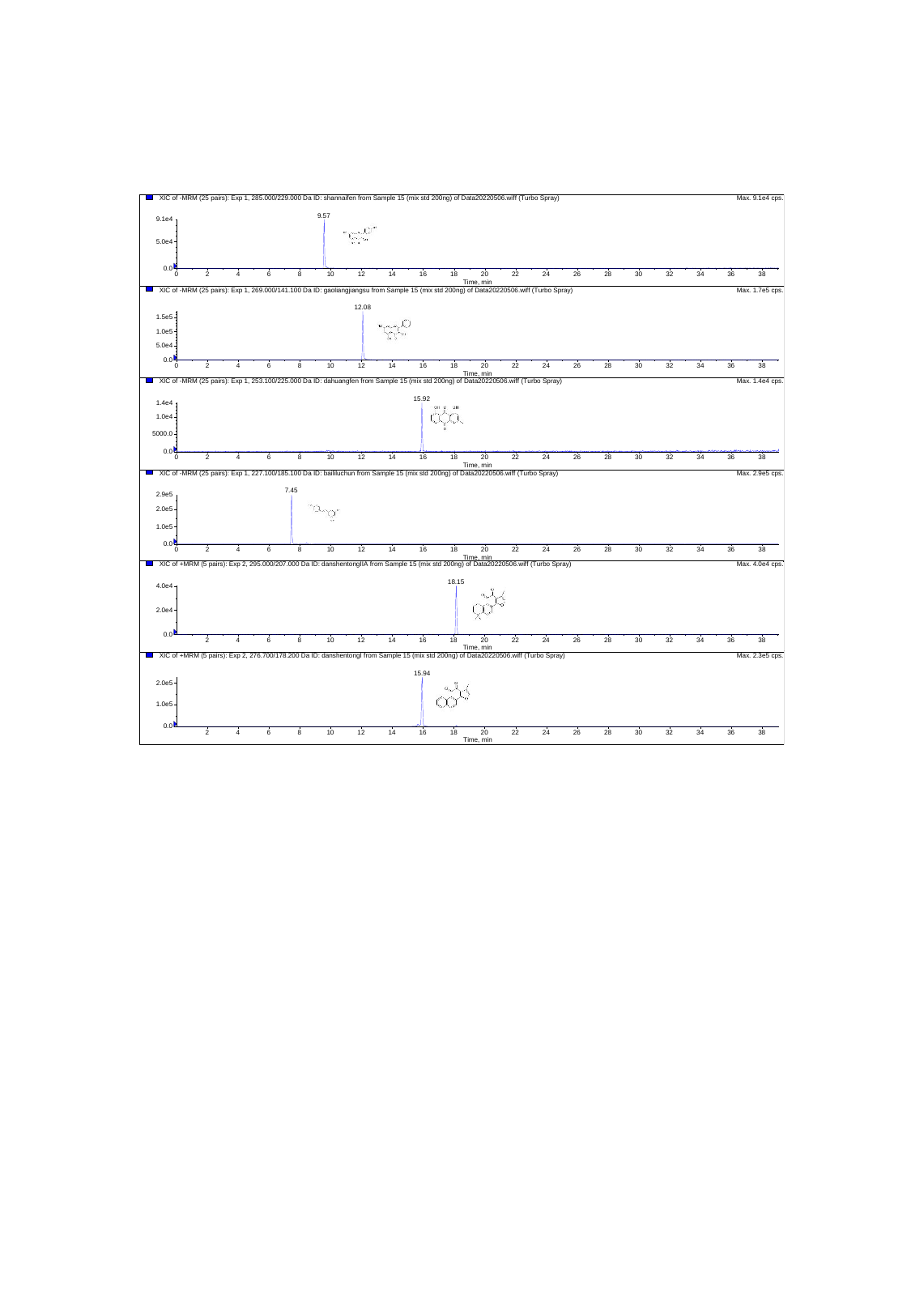

## Slide 9
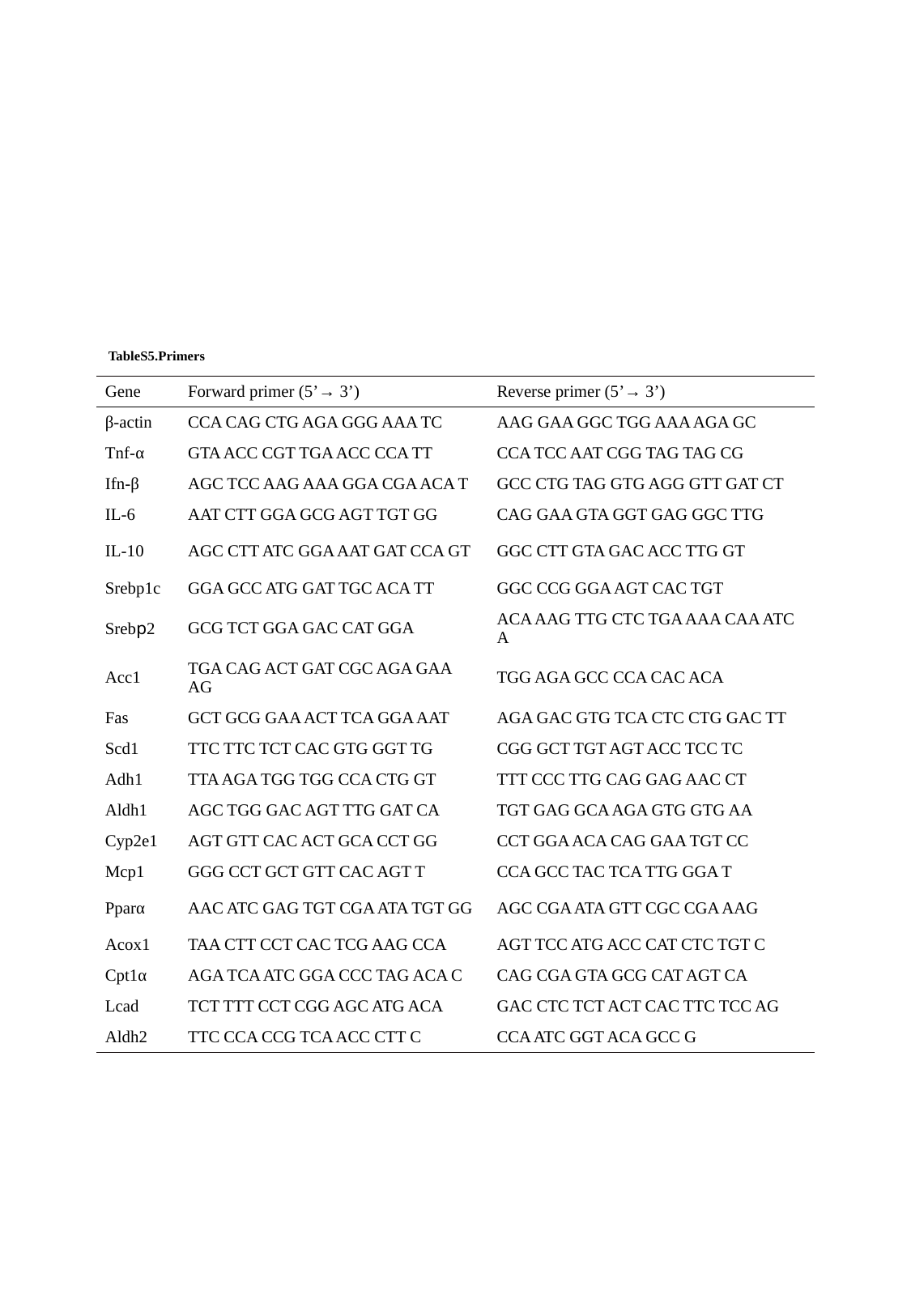

TableS5.Primers
| Gene | Forward primer (5’→ 3’) | Reverse primer (5’→ 3’) |
| --- | --- | --- |
| β-actin | CCA CAG CTG AGA GGG AAA TC | AAG GAA GGC TGG AAA AGA GC |
| Tnf-α | GTA ACC CGT TGA ACC CCA TT | CCA TCC AAT CGG TAG TAG CG |
| Ifn-β | AGC TCC AAG AAA GGA CGA ACA T | GCC CTG TAG GTG AGG GTT GAT CT |
| IL-6 | AAT CTT GGA GCG AGT TGT GG | CAG GAA GTA GGT GAG GGC TTG |
| IL-10 | AGC CTT ATC GGA AAT GAT CCA GT | GGC CTT GTA GAC ACC TTG GT |
| Srebp1c | GGA GCC ATG GAT TGC ACA TT | GGC CCG GGA AGT CAC TGT |
| Srebp2 | GCG TCT GGA GAC CAT GGA | ACA AAG TTG CTC TGA AAA CAA ATC A |
| Acc1 | TGA CAG ACT GAT CGC AGA GAA AG | TGG AGA GCC CCA CAC ACA |
| Fas | GCT GCG GAA ACT TCA GGA AAT | AGA GAC GTG TCA CTC CTG GAC TT |
| Scd1 | TTC TTC TCT CAC GTG GGT TG | CGG GCT TGT AGT ACC TCC TC |
| Adh1 | TTA AGA TGG TGG CCA CTG GT | TTT CCC TTG CAG GAG AAC CT |
| Aldh1 | AGC TGG GAC AGT TTG GAT CA | TGT GAG GCA AGA GTG GTG AA |
| Cyp2e1 | AGT GTT CAC ACT GCA CCT GG | CCT GGA ACA CAG GAA TGT CC |
| Mcp1 | GGG CCT GCT GTT CAC AGT T | CCA GCC TAC TCA TTG GGA T |
| Pparα | AAC ATC GAG TGT CGA ATA TGT GG | AGC CGA ATA GTT CGC CGA AAG |
| Acox1 | TAA CTT CCT CAC TCG AAG CCA | AGT TCC ATG ACC CAT CTC TGT C |
| Cpt1α | AGA TCA ATC GGA CCC TAG ACA C | CAG CGA GTA GCG CAT AGT CA |
| Lcad | TCT TTT CCT CGG AGC ATG ACA | GAC CTC TCT ACT CAC TTC TCC AG |
| Aldh2 | TTC CCA CCG TCA ACC CTT C | CCA ATC GGT ACA GCC G |
